# The Hymenopteran Tree of Life: Evidence from Protein-Coding Genes and Objectively Aligned Ribosomal Data

**DOI:** 10.1371/journal.pone.0069344

**Published:** 2013-08-02

**Authors:** Seraina Klopfstein, Lars Vilhelmsen, John M. Heraty, Michael Sharkey, Fredrik Ronquist

**Affiliations:** 1 Department of Biodiversity Informatics, Swedish Museum of Natural History, Stockholm, Sweden; 2 Natural History Museum of Denmark, Copenhagen, Denmark; 3 Department of Entomology, University of California Riverside, California, United States of America; 4 Department of Entomology, University of Kentucky, Lexington, Kentucky, United States of America; BiK-F Biodiversity and Climate Research Center, Germany

## Abstract

Previous molecular analyses of higher hymenopteran relationships have largely been based on subjectively aligned ribosomal sequences (18S and 28S). Here, we reanalyze the 18S and 28S data (unaligned about 4.4 kb) using an objective and a semi-objective alignment approach, based on MAFFT and BAli-Phy, respectively. Furthermore, we present the first analyses of a substantial protein-coding data set (4.6 kb from one mitochondrial and four nuclear genes). Our results indicate that previous studies may have suffered from inflated support values due to subjective alignment of the ribosomal sequences, but apparently not from significant biases. The protein data provide independent confirmation of several earlier results, including the monophyly of non-xyelid hymenopterans, Pamphilioidea + Unicalcarida, Unicalcarida, Vespina, Apocrita, Proctotrupomorpha and core Proctotrupomorpha. The protein data confirm that Aculeata are nested within a paraphyletic Evaniomorpha, but cast doubt on the monophyly of Evanioidea. Combining the available morphological, ribosomal and protein-coding data, we examine the total-evidence signal as well as congruence and conflict among the three data sources. Despite an emerging consensus on many higher-level hymenopteran relationships, several problems remain unresolved or contentious, including rooting of the hymenopteran tree, relationships of the woodwasps, placement of Stephanoidea and Ceraphronoidea, and the sister group of Aculeata.

## Introduction

The Hymenoptera (sawflies, wasps, bees and ants) are one of the four largest insect orders, with more than 146,000 described species [1] (J.T. Huber, personal communication). The oldest fossils belong to the family Xyelidae and date back to the middle Triassic (about 235 Ma) [2], but recent age estimates based on molecular data suggest a much earlier origin in the late Carboniferous (about 311 Ma) [3,4]. Hymenoptera assume a wide range of different life styles, from phytophagous to parasitic and predatory [1,5], occupy a wide range of ecological niches, and have undergone several transitions to eusociality [6,7]. Most species live as parasitoids of other insect larvae and thus fulfill a vital role in most terrestrial ecosystems, and many aculeates are economically important pollinators or predators. Despite their ecological and economic importance, especially the parasitic hymenopterans are one of the most severely understudied insect groups, with large regions of the world virtually unexplored, and undescribed species discovered at a regular pace even in well-studied faunas in the Western Palearctic and Nearctic [8]. Conservative estimates suggest that over 600,000 species of Hymenoptera may exist [9], although much higher numbers of 1-2.5 million species have been proposed [10,11].

The history of hymenopteran phylogenetic research dates back to pre-cladistic times, when the traditional division into the *Symphyta* (sawflies and woodwasps, without a wasp waist) and Apocrita (hymenopterans with a wasp waist) was established, as was the paraphily of the former with respect to the latter [12]. Apocritans were further divided into the Parasitica (parasitoid wasps) and Aculeata (stinging wasps), with the latter believed to be nested within the former. Rasnitsyn, in a series of seminal papers examining the morphology of both recent and fossil taxa [2,13] (and references there-in), proposed a very influential phylogenetic hypothesis. One of the most innovative aspects of this hypothesis was the division of the Apocrita into four clades, the Evaniomorpha, Proctotrupomorpha, Ichneumonoidea (‘Ichneumonomorpha’) and Aculeata (‘Vespomorpha’), only the last two of which had been recognized previously. Rasnitsyn was also the first to provide convincing evidence for the monophyly of ‘Vespina’, consisting of the sawfly family Orussidae and the Apocrita. However, Rasnitsyn never provided an explicit quantitative analysis, and a later attempt to specify the character observations and subject them to cladistics analysis [14] indicated that there was little objective support for the proposed groupings in the Apocrita.

Since then, several morphological and early molecular studies have improved our understanding of hymenopteran relationships while leaving many questions open. Sharkey [12] summarized earlier attempts to reconstruct the hymenopteran tree of life, setting the stage for a concerted effort of many international specialists collaborating under the Hymenoptera Tree of Life project (HymAToL). Three papers on higher-level hymenopteran relationships stemming from this project have recently been published, relying on morphology [15], molecular data [16], and both [17]. Vilhelmsen et al. [15] described 273 morphological characters from mesosomal anatomy, scored them for 89 species across the hymenopteran tree, and assessed their phylogenetic information content. Heraty et al. [16] analyzed approx. 6.2 kb of molecular sequences from four markers: the ribosomal 18S and 28S, the mitochondrial cytochrome oxidase 1 (CO1) and one copy of the nuclear elongation factor 1-α. They used both parsimony and statistical approaches. Sharkey et al. [17] combined the molecular dataset, Vilhelmsen et al’s [15] mesosomal characters, and 115 additional morphological characters from other parts of the body into a total-evidence dataset which they analyzed under the parsimony criterion.

Briefly, these studies show that morphological data resolve part of the basal sawfly grade but contain little information about relationships above the superfamily level in the Apocrita. The molecular data, in contrast, shed considerable light on apocritan relationships. For instance, they support the monophyly of Proctotrupomorpha, while showing that the Aculeata are nested within a paraphyletic Evaniomorpha. They also corroborate the monophyly of the much discussed Evanioidea (including Gasteruptiidae, Aulacidae and Evaniidae), and identify several novel groupings, such as the ‘core Proctotrupomorpha’ (Proctotrupomorpha without Cynipoidea and Platygastroidea), the Diaprioidea (Diapriidae, Monomachidae and Maamingidae), the ‘core Proctotrupoidea’ (Proctotrupoidea without Diaprioidea), and a clade consisting of Trigonaloidea + Megalyroidea. At the same time, the molecular data leave many parts of the apocritan tree unresolved, in particular relationships within Aculeata and Evaniomorpha. More disturbingly, they also suggest groupings that conflict strongly with morphology-based conclusions on sawfly relationships. In particular, they fail to support the established consensus view on woodwasp relationships and, depending on alignment, even fail to support the monophyly of Apocrita itself, placing the Orussoidea among Evaniomorpha lineages.

One of the major problems in interpreting the molecular results is that they are based to a large extent on ribosomal data. The ribosomal sequences (18S and 28S) comprise almost three quarters of the HymAToL data, and an even larger fraction of the phylogenetically informative sites. Ribosomal sequences are challenging to align correctly, especially at the evolutionary distances involved in higher hymenopteran phylogeny, and all currently available methods involve some compromises. Heraty et al. [16] employed two approaches, a by-eye alignment and an alignment based on predicted secondary structure; Sharkey et al. [17] chose to use the former. Both methods rely on human judgment and hence the results may have been influenced by preconceived notions of phylogenetic relationships. As evidenced by the differences between the results based on the by-eye and secondary-structure alignments [16], the alignment method can strongly affect phylogenetic inference.

One way to remove potential alignment bias from the equation is to align the ribosomal sequences using methods that do not involve subjective human input. Another possibility is to infer relationships based entirely on easily aligned protein-coding sequences, but until now there have not been enough protein-coding data available. In this paper, we explore both tactics. First, we explore objective alignment of the ribosomal data. Ideally, the alignment should be based on models including nucleotide substitutions as well as insertion and deletion events, and phylogenetic inference should accommodate alignment uncertainty. In principle, such methods are available in a Bayesian framework [18,19], but they are still too computationally expensive to be applied to the HymAToL data. Instead, we use a two-step approach in which we obtain a ribosomal alignment without or with very little subjective human input first and then subject it to analysis using standard methods. Specifically, we use two methods for obtaining the ribosomal alignments: i) a fully objective, iterative approach using MAFFT [20]; and ii) a semi-objective Bayesian approach based on an explicit model of indel evolution, as implemented in the program BAli-Phy [21], applied to subalignments that are then pieced together. Second, we add three nuclear protein-coding genes to the HymAToL dataset: RNA polymerase II, the carbamoyl phosphate synthase domain of CAD, and the F1 copy of elongation factor 1-α. This allows us for the first time to infer higher relationships across the Hymenoptera based entirely on protein-coding data (4.6 kb from five markers). Finally, in order to identify the origins of different, sometimes conflicting, phylogenetic signals in the resulting data, we conducted in-depth analyses of the different data partitions separately and combined in a fully stochastic, Bayesian framework.

## Materials and Methods

### Taxon sampling and molecular methods

Our taxon sampling is largely based on the HymAToL sampling as described in Heraty et al. [16] and Sharkey et al. [17], with minor modifications. While excluding some of the aculeate taxa with low gene coverage that were over-represented in the data matrix, we added a representative of an additional family, the Megalodontesidae (Pamphilioidea). In total, we included 110 hymenopteran species covering 66 families and all 22 superfamilies [12], and 27 outgroup taxa (Table 1).

**Table 1:** Taxon sampling and Genbank accession numbers.

**Taxa**				**GenBank Accession Numbers**					
			**18S**	**28S**	**COI**	**EF1α-F1**	**EF1α-F2**	**POL**	**CAD**
Odonata	composite taxon^a^		FN3561661	FJ5965682	EF1767213	(missing)	AY5802114	AB5968995	(missing)
Orthoptera									
	Acrididae	several genera	AY859547	AY859546	EU370925	(missing)	AB583233	AB596906	(missing)
	Grylloidea	several genera	AY521869	AY859544	AF514693	(missing)	AB583232	AB596908	(missing)
	Stenopelmatidae	*Stenopelmatus* sp.	AY121145	AY125285	EF030116	(missing)	(missing)	(missing)	(missing)
Dermaptera	composite taxon		AY5218406	EU4268767	HM385637	(missing)	AY3054647	AY3055627	(missing)
Thysanoptera	composite taxon		AY6304458	AY5233848	GU3930239	(missing)	AY827479^10^	AB5969169	GQ2655888
Hemiptera	composite taxon		LHU0647611	DQ13358412	AY253038^13^, AY744838^14^		HP42935715	AB59691916	XM00194360017
Neuroptera	composite taxon		AF42379018	AY52179418	FJ85990619	(missing)	JQ519512^20^	AB59692721	KC213148^20^
*Megaloptera*	composite taxon		AY52186422	AY52179322	AY75051923	(missing)	HM15672122	AB59692524	EU86015422
Rhaphidioptera									
	Inocellidae	*Negha* sp.	AY521865	AY521795	EU839744	(missing)	(missing)	(missing)	EU860130
	Raphidiidae	Raphidiidae sp.	GU169690	GU169693	GU169696	(missing)	EU414713	(missing)	(missing)
Mecoptera									
	Panorpidae	*Panorpa* sp. (composite)	GU169691	GU169694	GU169697	(missing)	AF423866	AB596933	GQ265595
	Bittacidae	*Bittacus* sp. (composite)	AF286290	AF423933	EF050551	(missing)	AF423822	(missing)	GQ265603
Coleoptera									
	Belidae	*Oxycraspeduscribricollis* (Blanchard)	FJ867778	FJ867698	FJ867811	(missing)	FJ867881	(missing)	(missing)
	Scirtidae	several genera	GU591990	GU591989	NC011320	(missing)	(missing)	(missing)	(missing)
	Dytiscidae	several genera	GU591992	GU591991	FN263054	(missing)	FN256352	EU677586	EU677529
	Carabidae	*Bembidion* sp. (composite)	GQ503348	GQ503347	GU347089	(missing)	GQ503346	EU677593	EF649423
	Myxophaga	composite taxon	GU59199325	GU59199425	GQ50334226	(missing)	GQ50334526	HM156727^27^	HM156726^27^
	Archostemata	composite taxon	EU797411^28^	GU591995^29,30^	EU839762^31^	(missing)	GQ503344^32^	EU677579^32^	EU677525^32^
Lepidoptera									
	Cossidae	several genera	AF423783	AY521785	GU090140	(missing)	GU829379	(missing)	GQ283590
	Micropterigidae	*Micropterix* sp. (composite)	GU169692	GU169695	HQ200895	(missing)	GU828950, GU829241	(missing)	GU828116
Trichoptera									
	Hydropsychidae	*Hydropsyche* sp. (composite)	AF286291	AF338267	FN179145	(missing)	FM998455	FN178740	FN178964
Diptera									
	Deuterophlebiidae	*Deuterophlebia* *coloradensis* Pennak	FJ040539	FJ040539	GQ465781	(missing)	(missing)	(missing)	FJ040594
	Ptychopteridae	*Ptychopteraquadrifasciata* Say	FJ040542	GQ465777	GQ465782	(missing)	GQ465785	(missing)	FJ040598
	Tipulidae	*Tipulaabdominalis* Say (composite)	FJ040553	GQ465778	AY165639	(missing)	GQ465786	(missing)	GQ265584
	Stratiomyidae	*Hermetiaillucens* L.	DQ168754	GQ465779	GQ465783	(missing)	GQ465787	(missing)	(missing)
	Muscidae	*Musca* *domestica* L.	DQ656974	GQ465780	AF104622	(missing)	DQ657113	(missing)	AY280689
Hymenoptera									
Apoidea									
	Ampulicidae	*Ampulex* *compressa* (Fabricius)	GQ410619	GQ374726	GQ374639	JQ519513	JQ519593	(missing)	KC213149
	Apidae	*Apis mellifera* Linnaeus	AY703484	AY703551	FJ582090, AF250946	X52884, X52885	AF015267	KC213058	KC213150
		*Hesperapis* *regularis* (Cresson)	AY995665	†	GQ374630	(missing)	AY585151	KC213059	KC213151
	Crabronidae	*Pisonchilense* Spinola	GQ410608	GQ374715	GQ374629	JQ519514	JQ519595	KC213060	KC213152
	Sphecidae	*Stangeella* *cyaniventris* (Guérin-Ménevill	GQ410616	GQ374723	GQ374637	JQ519515	JQ519596	KC213061	KC213153
Cephoidea									
	Cephidae	*Cephus* *pygmeus* (Linnaeus) / *C* *. nigrinus* (Thomson)	GQ410588	GQ374695	EF032228	(missing)	JQ519597	KC213062	KC213154
		*Hartigiatrimaculata* (Say)	GQ410589	GQ374696	EF032230	JQ519516	JQ519598	KC213063	KC213155
Ceraphronoidea									
	Ceraphronidae	*Ceraphron* *bispinosus* (Nees), *Ceraphron* sp.	GQ410626	GQ374733	GQ374642	(missing)	JQ519599	KC213064	KC213156
	Megaspilidae	*Lagynodes* sp.	GQ410624	GQ374731	(missing)	JQ519517	JQ519600	KC213065	KC213157
		*Megaspilus* *fuscipennis* (Ashmead)	GQ410625	GQ374732	(missing)	JQ519518	JQ519601	KC213066	KC213158
Chalcidoidea									
	Aphelinidae	*Coccobius* *fulvus* (Compere & Annecke)	GQ410673	GQ374780	GQ374675	(missing)	(missing)	(missing)	(missing)
		*Coccophagusrusti* Compere	GQ410674	GQ374781	GQ374676	JQ519519	JQ519602	KC213067	(missing)
	Calesinae	*Calesnoacki* Howard	GQ410670	GQ374777	(missing)	(missing)	JQ519603	KC213068	(missing)
	Chalcididae	*Acanthochalcis* *nigricans* Cameron	GQ410679	GQ374786	GQ374680	(missing)	JQ519604	(missing)	(missing)
	Eucharitidae	*Psilocharis* *afra* Heraty	GQ410680	GQ374787	KC213237	JQ519520	JQ519605	KC213069	(missing)
	Eulophidae	*Cirrospilus* *coachellae* Gates	GQ410672	GQ374779	GQ374674	JQ519521	JQ519606	KC213070	KC213159
	Eurytomidae	*Eurytoma* *gigantea* Walsh	GQ410671	GQ374778	GQ374673	(missing)	JQ519607	KC213071	(missing)
	Mymaridae	*Australomymar* sp.	GQ410668	GQ374775	GQ374671	JQ519522	(missing)	KC213072	KC213160
		*Gonatocerusashmeadi* Girault, *Gonatorcerus* sp.	GQ410667	GQ374774	DQ328644	(missing)	JQ519608	KC213073	KC213161
	Pteromalidae	*Cleonymus* sp.	GQ410678	GQ374785	GQ374679	(missing)	JQ519609	KC213074	KC213162
		*Nasonia* *vitripennis* Walker	GQ410677	GQ374784	GQ374678	NC015867	JQ519610	KC213075	KC213163
	Rotoitidae	*Chiloe micropteron* Gibson & Huber	GQ410669	GQ374776	GQ374672	(missing)	JQ519611	(missing)	(missing)
	Tetracampidae	*Foersterella* *reptans* (Nees)	GQ410675	GQ374782	KC213238	(missing)	JQ519612	KC213076	KC213164
	Torymidae	*Megastigmus* *transvaalensis* (Hussey)	GQ410676	GQ374783	GQ374677	JQ519523	JQ519613	KC213077	KC213165
Chrysidoidea									
	Bethylidae	*Cephalonomiastephanoderis* Betrem	GQ410610	GQ374717	GQ374632	JQ519524	JQ519614	KC213078	KC213166
	Chrysididae	*Chrysiscembricola* Krombein	GQ410611	GQ374718	GQ374633	JQ519525	(missing)	(missing)	KC213167
	Plumariidae	*Myrmecopterina* sp.	GQ410618	GQ374725	KC213239	JQ519526	(missing)	KC213079	KC213168
	Scolebythidae	*Scolebythusmadecassus* Evans	GQ410609	GQ374716	GQ374631	JQ519527	JQ519615	KC213080	KC213169
Cynipoidea									
	Cynipidae	*Diplolepis* sp.	GQ410647	GQ374754	GQ374659	JQ519528	JQ519616	KC213081	(missing)
		*Periclistus* sp.	GQ410648	GQ374755	AF395181	JQ519529	JQ519617	KC213082	KC213170
	Figitidae	*Anacharis* sp.	GQ410651	GQ374758	(missing)	JQ519530	JQ519618	KC213083	KC213171
		*Melanips* sp.	GQ410649	GQ374756	GQ374660	JQ519531	JQ519619	KC213084	KC213172
		*Parnips* *nigripes* (Barbotin)	GQ410650	GQ374757	GQ374661	JQ519532	JQ519620	KC213085	KC213173
	Ibaliidae	*Ibalia* sp.	GQ410645	GQ374752	GQ374657	JQ519533	JQ519621	KC213086	KC213174
	Liopteridae	*Paramblynotus* sp.	GQ410646	GQ374753	GQ374658	JQ519534	JQ519622	KC213087	KC213175
Diaprioidea									
	Diapriidae	*Belyta* sp.	GQ410663	GQ374770	(missing)	JQ519535	JQ519623	KC213088	KC213176
		*Ismarus* sp.	GQ410662	GQ374769	GQ374668	JQ519536	(missing)	KC213089	KC213177
		*Pantolytomyia* *ferruginea* Dodd	GQ410660	GQ374767	GQ374666	JQ519537	JQ519624	KC213090	KC213178
		*Poecilopsilus* sp.	GQ410661	GQ374768	GQ374667	JQ519538	JQ519625	(missing)	KC213179
	Maamingidae	*Maamingamarrisi* Early et al.*, Maaminga* sp.	GQ410664	GQ374771	GQ374669	JQ519539	JQ519626	KC213091	KC213180
	Monomachidae	*Monomachus* sp.	GQ410652	GQ374759	GQ374662	JQ519540	JQ519627	KC213092	KC213181
Evanioidea									
	Aulacidae	*Aulacusimpolitus* Smith	GQ410638	GQ374745	GQ374652	JQ519541	JQ519628	(missing)	KC213182
		*Pristaulacusstrangaliae* Rohwer	GQ410635	GQ374742	GQ374649	JQ519542	JQ519629	KC213093	KC213183
	Evaniidae	*Brachygaster* *minuta* (Olivier)	GQ410634	GQ374741	AY800156	JQ519543	(missing)	KC213094	KC213184
		*Evaniaalbofacialis* Cameron	GQ410632	GQ374739	GQ374647	JQ519544	(missing)	(missing)	KC213185
		*Evaniellasemaeoda* Bradley	GQ410633	GQ374740	GQ374648	JQ519545	JQ519630	KC213095	KC213186
	Gasteruptiidae	*Gasteruption* sp*.*	GQ410636	GQ374743	GQ374650	JQ519546	JQ519631	(missing)	KC213187
		*Pseudofoenus* sp*.*	GQ410637	GQ374744	GQ374651	JQ519547	JQ519632	KC213096	KC213188
Ichneumonoidea									
	Braconidae	*Aleiodes* *terminalis* Cresson, *A* *. dissector* (Nees)	GQ410603	GQ374710	EF115472	JQ519548	JQ519633	KC213097	KC213189
		*Doryctes* *erythromelas* (Brullé), *Doryctes* sp.	GQ410602	GQ374709	GQ374627	JQ519549	JQ519634	KC213098	KC213190
		*Rhysipolis* sp.	GQ410601	GQ374708	GQ374626	JQ519550	JQ519635	KC213099	KC213191
		*Wroughtonialigator* (Say)	GQ410600	GQ374707	GQ374625	JQ519551	JQ519636	(missing)	KC213192
	Ichneumonidae	*Dusona* *egregia* (Viereck)	GQ410597	GQ374704	AF146682	JQ519552	JQ519637	KC213100	KC213193
		*Labenagrallator* (Say)	GQ410595	GQ374702	GQ374622	(missing)	JQ519638	KC213101	KC213194
		*Lymeon* *orbus* (Say)	GQ410599	GQ374706	GQ374624	JQ519553	JQ519639	KC213102	KC213195
		*Pimpla* *aequalis* Provancher	GQ410598	GQ374705	AF146681	(missing)	JQ519640	KC213103	KC213196
		*Zagryphus* *nasutus* (Cresson), *Zagryphus* sp.	GQ410596	GQ374703	GQ374623	JQ519554	JQ519641	KC213104	KC213197
Megalyroidea									
	Megalyridae	*Megalyra* sp.	GQ410629	GQ374736	GQ374645	(missing)	JQ519642	KC213105	KC213198
Mymarommatoidea									
	Mymarommatidae	*Mymaromella* *mira* Girault	GQ410666	GQ374773	KC213240	(missing)	(missing)	KC213106	(missing)
		*Mymarommaanomalum* (Blood & Kryger)	GQ410665	GQ374772	GQ374670	(missing)	JQ519643	KC213107	(missing)
Orussoidea									
	Orussidae	*Orussobaius* *wilsoni* Benson	GQ410607	GQ374714	(missing)	(missing)	(missing)	(missing)	(missing)
		*Orussusabietinus* (Scopoli)	GQ410604	GQ374711	EF032236	JQ519555	JQ519644	KC213108	KC213199
		*Orussus* *occidentalis* (Cresson)	GQ410605	GQ374712	GQ374628	JQ519556	JQ519645	(missing)	KC213200
Pamphilioidea									
	Megalodontesidae	*Megalodontes* *cephalotes* (Fabricius)	AY621138	EF032260	EF032227	JQ519557	JQ519646	KC213109	KC213201
	Pamphiliidae	*Cephalcia* cf. *abietis* (Linnaeus)	GQ410587	GQ374694	EF032225	JQ519558	JQ519647	KC213110	(missing)
		*Onycholydaamplecta* (Fabricius)	GQ410586	GQ374693	EF032223	JQ519559	JQ519648	KC213111	KC213202
Platygastroidea									
	Platygastridae	*Isostasius* sp.	GQ410644	GQ374751	KC213241	(missing)	(missing)	(missing)	(missing)
		*Platygaster* sp.	GQ410641	GQ374748	GQ374654	(missing)	JQ519649	(missing)	KC213203
		*Proplatygaster* sp.	GQ410643	GQ374750	GQ374656	(missing)	(missing)	(missing)	(missing)
	Scelionidae (*s.str.*)	*Archaeoteleiamellea*	GQ410639	GQ374746	GQ374653	JQ519560	JQ519650	KC213112	KC213204
		*Telenomus* sp.	GQ410642	GQ374749	GQ374655	JQ519561	JQ519651	KC213113	KC213205
Proctotrupoidea									
	Heloridae	*Helorus* sp.	GQ410653	GQ374760	GQ374663	JQ519562	JQ519652	KC213114	KC213206
	Pelecinidae	*Pelecinuspolyturator* (Drury)	GQ410655	GQ374762	GQ374664	JQ519563	JQ519653	KC213115	KC213207
	Proctotrupidae	*Austroserphus* sp.	GQ410654	GQ374761	(missing)	JQ519564	JQ519654	KC213116	KC213208
		*Exallonyx* sp.	GQ410656	GQ374763	(missing)	JQ519565	JQ519655	KC213117	KC213209
		*Proctotrupes* sp.	GQ410657	GQ374764	(missing)	JQ519566	(missing)	KC213118	(missing)
	Roproniidae	*Roproniagarmani* Ashmead	GQ410659	GQ374766	GQ374665	(missing)	GQ410745	KC213119	(missing)
	Vanhornidae	*Vanhorniaeucnemidarum* Crawford	GQ410658	GQ374765	DQ302100	(missing)	JQ519656	KC213120	KC213210
Siricoidea									
	Anaxyelidae	*Syntexislibocedrii* Rohwer	GQ410594	GQ374701	EF032234	(missing)	JQ519657	KC213121	(missing)
	Siricidae	*Sirex* sp.	GQ410593	GQ374700	GQ374621	JQ519567	JQ519658	KC213122	KC213211
		*Tremex* *columba* (Linnaeus), *Tremex* sp.	GQ410592	GQ374699	EF032233	JQ519568	JQ519659	KC213123	KC213212
Stephanoidea									
	Stephanidae	*Megischus* sp.	GQ410630	GQ374737	GQ374646	JQ519569	JQ519660	KC213124	KC213213
		*Schlettereriuscinctipes* (Cresson)	GQ410631	GQ374738	EF032237	JQ519570	(missing)	KC213125	KC213214
Tenthredinoidea									
	Argidae	*Atomacera* *debilis* Say, *Arge* *nigripes* (Retzius)	GQ410580	GQ374687	GQ374618	JQ519571	JQ519661	KC213126	KC213215
		*Sterictiphora* *furcata* (Villers)	GQ410578	GQ374685	EF032222	JQ519572	JQ519662	(missing)	KC213216
	Blasticotomidae	*Runaria* *reducta* Malaise, *R* *. flavipes* Takeuchi	GQ410581	GQ374688	EF032212	JQ519573	JQ519663	KC213127	(missing)
	Cimbicidae	*Corynis* *crassicornis* (Rossi)	GQ410577	GQ374684	EF032220	JQ519574	JQ519664	KC213128	KC213217
	Diprionidae	*Monoctenus* *juniperi* (Linnaeus)	GQ410582	GQ374689	EF032278	JQ519575	JQ519665	KC213129	KC213218
	Pergidae	*Decameria* *similis* (Enderlein)	GQ410579	GQ374686	GQ374617	(missing)	(missing)	(missing)	(missing)
		*Heteroperreyiahubrichi* Malaise	GQ410585	GQ374692	GQ374620	JQ519576	JQ519666	KC213130	(missing)
	Tenthredinidae	*Athaliarosae* (Linnaeus)	GQ410576	GQ374683	GQ374616	JQ519577	JQ519667	KC213131	KC213219
		*Notofenusasurosa* (Konow)	GQ410584	GQ374691	(missing)	JQ519578	JQ519668	KC213132	KC213220
		*Tenthredo* *campestris* Linnaeus	GQ410583	GQ374690	GQ374619	(missing)	JQ519669	KC213133	KC213221
Trigonaloidea									
	Trigonalidae	*Orthogonalys* *pulchella* (Cresson)	GQ410628	GQ374735	GQ374644	JQ519579	JQ519670	KC213134	KC213222
		*Taeniogonalys* *gundlachii* (Cresson)	GQ410627	GQ374734	GQ374643	JQ519580	JQ519671	KC213135	KC213223
Vespoidea									
	Bradynobaenidae	*Chyphotesmellipes* (Blake), *Chyphotes* sp.	AY703485	AY703552	DQ353285	JQ519581	JQ519672	KC213136	KC213224
	Formicidae	*Formica moki* Wheeler, *Formica* sp.	AY703493	AY703560	AF398151	JQ519582	JQ519673	(missing)	KC213225
		*Myrmica* *tahoensis* Weber	AY703495	AY703562	DQ353360	AY363040	(missing)	(missing)	(missing)
		*Paraponera* *clavata* (Fabricius)	AY703489	AY703556	GQ374640	JQ519583	JQ519674	KC213137	KC213226
	Mutillidae	*Dasymutilla* *aureola* (Cresson), *D* *. vesta* (Cresson)	GQ410621	GQ374728	EU567203	JQ519584	JQ519675	KC213138	KC213227
	Pompilidae	*Aporus* *niger* (Cresson)	GQ410615	GQ374722	GQ374636	JQ519585	JQ519676	KC213139	KC213228
	Rhopalosomatidae	*Rhopalosomanearcticum* Brues	GQ410617	GQ374724	GQ374638	JQ519586	JQ519677	KC213140	KC213229
	Sapygidae	*Sapyga* *pumila* Cresson	GQ410612	GQ374719	GQ374634	JQ519587	JQ519678	KC213141	KC213230
	Scoliidae	*Scolia* *verticalis* Fabricius	EF012932	EF013060	GQ374641	JQ519588	JQ519679	KC213142	KC213231
	Tiphiidae	*Colocistis* cf. *sulcatus* (M. & K.), *Brachycistis* sp.	GQ410623	GQ374730	KC213242	(missing)	(missing)	KC213143	KC213232
	Vespidae	*Metapolybiacingulata* (Fabricius)	GQ410613	GQ374720	GQ374635	JQ519589	JQ519680	KC213144	KC213233
Xiphydrioidea									
	Xiphydriidae	*Derecyrta* *circularis* Smith	GQ410591	GQ374698	(missing)	(missing)	(missing)	(missing)	(missing)
		*Xiphydriaprolongata* (Geoffroy)	GQ410590	GQ374697	EF032235	JQ519590	JQ519681	KC213145	KC213234
Xyeloidea									
	Xyelidae	*Macroxyela* *ferruginea* (Say)	GQ410574	GQ374681	EF032211	JQ519591	JQ519682	KC213146	KC213235
		*Xyelajulii* (Brebisson)	GQ410575	GQ374682	EF032210	JQ519592	JQ519683	KC213147	KC213236

^†^is a combination of AY654456, AY654457, and AY654522.

^a^ Composite taxa comprised of sequences from more than one taxons follows: Corduliidae ^1^: *Somatochloragraeseri* Selys^2^, *Somatochlora* *alpestris* Selys; Coenagrionidae ^3^: *Erythrommanajas* Hansemann^4^, *Enallagma* *aspersum* (Hagen); Calopterygidae ^5^: *Mnais* *pruinosa* Selys; Spongiphoridae ^6^: *Auchenomusforcipatus* Ramamurthi; Forficulidae ^7^: *Forficula* *auricularia* L.; Thripidae ^8^: *Frankliniella* sp.^9^, *Thrips* sp.; Phlaeothripidae ^10^: *Kladothripsnicolsoni* McLeish, Chapman & Mound^11^; *Lygus* *hesperus* Knight; Phymatidae^12^: *Phymata* sp. (D1-6); Miridae ^13^: *Lyguselisus* (Van Duzee); Cixiidae ^14^: *Pintalia* *alta* Osborn (D7-10); Reduviidae ^15^: *Triatoma matogrossensis* Leite & Barbosa; Coreidae ^16^: *Anacanthocorisstriicornis* (Scott); Aphididae ^17^: *Acyrthosiphon pisum* Harris; Hemerobiidae ^18^: *Hemerobius* sp.; Mantispidae ^19^: *Ditaxis* *biseriata* (Westwood); Chrysopidae ^20^: *Chrysopaperla* (L.)^21^, *Chrysoperla* *nipponensis* (Okamoto); Sialidae ^22^: *Sialis* sp.; Corydalidae ^23^: *Nigronia* *fasciatus* (Walker)^24^; *Protohermesgrandis* (Thunberg); Lepiceridae ^25^: *Lepicerus* *inaequalis* Motschulsky; Sphaeriusidae ^26^: *Sphaerius* sp.; Hydroscaphidae ^27^: *Hydroscapha* *natans* LeConte; Cupedidae ^28^: *Prolixocupeslobiceps* (LeConte) (18S); ^29^P*. lobiceps*, D2-D5 (GU591995) and Ommatidae ^30^: *Tetraphalerusbruchi* Heller, D1 and D6-D10 (Maddison BToL, not yet deposited), and Cupedidae ^31^: *Priacma* *serrata* LeConte^32^; *Tenomerga* sp.

The previous data matrix from the HymAToL project encompassed, unaligned, about 1,400 bp of 18S rRNA (by-eye alignment: 2,014 bp, secondary structure alignment: 1,860 bp), about 3,000 bp of 28S rRNA (by-eye alignment: 4,681 bp, secondary structure alignment: 3,252 bp; both after exclusion of unreliably aligned portions), 770 bp of CO1 mtDNA, and 1,040 bp of the coding region of the F2 copy of elongation factor 1-α (EF1α-F2). To these four markers, we added sequences from three nuclear, protein-coding genes: 990 bp of the carbamoylphosphate synthetase domain of the Conserved ATPase Domain (CAD), 800 bp of RNA polymerase II (POL), and 1,040 bp of the F1 copy of the elongation factor 1-α (EF1α-F1). The F1 and F2 copies of EF1α in Hymenoptera originate from a duplication event that took place before the radiation of the order and the two copies evolved independently since [22].

Laboratory protocols followed Heraty et al. [16] and Klopfstein and Ronquist [22]. The wide taxonomic scope of this study necessitated the use of a range of primer pairs for different taxonomic groups. While primers and protocols used for the EF1α-F1 sequences are given elsewhere [22], primers for CAD and POL are listed in Supplementary Table S1. Gene coverage was 84%, so on average six of the seven genes were sequenced per taxon. The 18S and 28S genes were sequenced for all taxa, CO1 for 92%, EF1α-F2 for 85%, EF1α-F1 for 61% (75% in Hymenoptera), POL for 76% and CAD for 80% of the taxa. Genbank accession numbers are given in Table 1.

### Multiple-sequence alignment

Protein-coding genes were aligned in Mega5 [23] after translation into amino acids. Few gaps were detected, and alignment was straightforward. Introns were identified by alignment against known coding regions from Genbank (Table 1) and their exact position conditioned on the presence of GT-AG splicing sites. Introns were not objectively alignable and were removed from all further analyses.

For the MAFFT alignment of the ribosomal sequences, we used the E-INS-i algorithm as available on the web server at http://mafft.cbrc.jp/alignment/server/ with all parameters at their default values [20]. This algorithm has been shown to be more accurate for difficult alignments than other iterative alignment procedures on a wide range of benchmarks, in several simulation studies [24,25], and also was the preferred alignment algorithm for ribosomal stem regions in analyses of Chalcidoidea [26].

As an alternative approach, we used the program BAli-Phy [19,21] with a model of indel evolution that takes branch lengths into account [27] to obtain MAP (maximum posterior probability) subalignments of subsets of taxa that were later pieced together into a complete alignment. We split our data in four different taxon sets. The first set included all outgroup taxa and one representative of each hymenopteran superfamily, the second set contained all remaining symphytan taxa, the third the species of Proctotrupomorpha, and the fourth the rest of the apocritan taxa. Each of these four taxon sets was then aligned separately in BAli-Phy under a GTR + Γ + I substitution model. In order to speed up convergence, we introduced multiple alignment constraints. To do so, we examined the secondary-structure alignment from Heraty et al. [16] for length-constant stem regions of at least length 10 bp, and fixed the alignment at a conserved base in the middle of each such stem. A total of 48 and 85 alignment constraints were invoked for 18S and 28S, respectively. Because the 28S alignment used too much memory to be run in a single analysis, we cut the alignment into two parts at one of the constraint points around the middle of the sequence, and ran it in two separate analyses. For all four taxon sets, which included from 16 to 33 taxa each, we ran four independent runs for seven days (the maximum period) at the National Supercomputer Center in Linköping, Sweden (NSC). Most of the runs did not reach the aspired topology convergence (the average standard deviation of split frequencies (ASDSF) between runs for the different taxon sets was 0.003-0.09 for 18S and 0.04-0.17 for 28S), but the sample of other parameters had reached convergence as judged from effective sample sizes > 100. The MAP alignments obtained from these runs were combined using OPAL [28]. First, we merged the outgroup and backbone taxa with *Symphyta* , then added the remaining Apocrita without Proctotrupomorpha, and finally merged all of these with Proctotrupomorpha. Alignment and polishing methods were set to “exact”, the distance type to “normalized alignment costs”, and the polishing approach to “random three-cut”. Nineteen of the 18S and 36 of the 28S sequences had missing parts, which were not sequenced. Because BAli-Phy relies on an explicit indel model of evolution and gaps thus become informative, these sequences had to be removed from the BAli-Phy analyses. We added these fragmentary sequences to the final BAli-Phy alignment using the “add” option in MAFFT [29].

### Data properties

**Figure 1:**
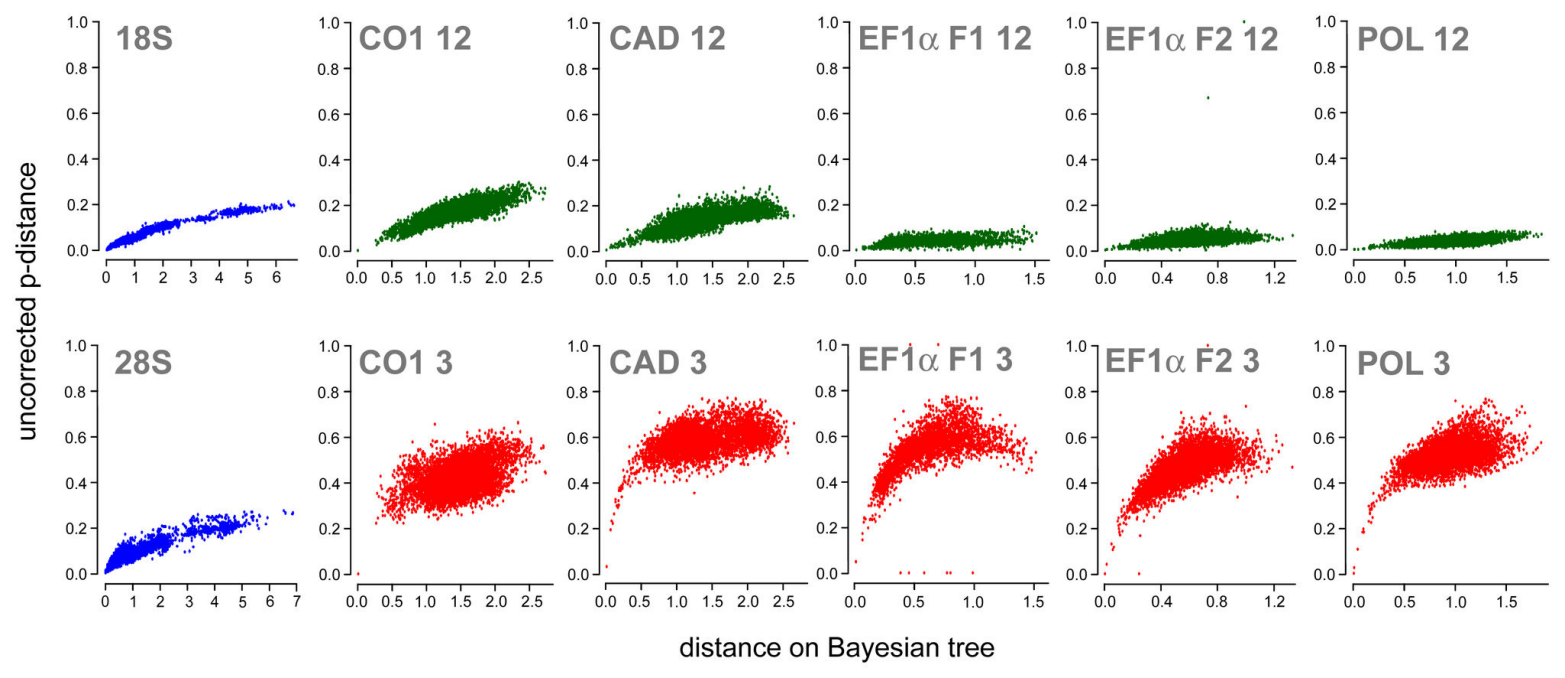
Saturation plots of the different genes and codon positions. Uncorrected p-distances are shown on the y-axis, while the x-axis represents the pairwise distances as inferred on the tree recovered from the single-gene analyses. “CO1 12” indicates the combined first and second codon position of the CO1 gene, and so forth.

The variation present in the different genes and gene partitions was examined using the “cstatus” command, and a basic test of non-stationarity of nucleotide composition was performed with the command “basefreqs” in PAUP* [30]. Saturation plots for each gene and for the third codon positions of protein-coding genes were produced by retrieving pairwise uncorrected p-distances in Mega 5 [23], and plotting them against inferred branch-length distances on the tree with the highest likelihood found during the Bayesian tree search based on the single genes (R script available from the first author on request). The third codon positions of all genes showed clear signs of saturation and non-stationarity (Table 2 and Figure 1), so we also analyzed our data after excluding them.

**Table 2:** Data properties.

**Gene/partition**	**#bp**	**#var**	**#pars**	**GC%**	**Stationarity**
18S^1^	2,027/2,310	959/1,003	659/610	50.0%	p>0.05
28S^1^	5418/10,557	3486/4,456	2,279/1,938	55.2%	p<0.001
CAD12	658	360	284	42.2%	p>0.05
CAD3	330	321	316	48.3%	p<0.001
POL 12	535	154	84	43.8%	p>0.05
POL 3	268	265	259	45.3%	p<0.001
EFF1 12	695	220	119	48.5%	p>0.05
EFF1 3	348	338	338	61.6%	p<0.001
EFF2 12	695	221	132	49.2%	p>0.05
EFF2 3	348	337	335	55.5%	p<0.001
CO1 12	526	324	264	38.0%	p<0.001
CO1 3	263	262	262	10.1%	p<0.001
Morphology	391	391	387		
Total^12^	12,111/17,533	7,247/8,261	5,331/4,941	49.6%	p<0.1
Total analyzed^13^	10,554/15,976	5,724/6,738	3,821/3,431	50.4%	p<0.001

^1^ Values for the ribosomal RNA are given both for the MAFFT and the BAli-Phy alignments. Unaligned sequences vary a lot in length between taxa, but are about 1,400 bp for 18S and about 3,000 for 28S.

^2^ molecular data combined, before exclusion of the third codon positions, without morphology

^3^ molecular data combined, after exclusion of the third codon positions, without morphology

In order to get a rough estimate of the performance of the different genes (or of their contribution to the final phylogenetic inference) and to assess the quality of the two alignment approaches for the rRNA partition, we compared the Bayesian tree samples obtained from the single-gene analyses and from an analysis of morphology alone (see below) to the protein-coding and total-evidence tree samples. As a measure of topological distance, we used ASDSF values as obtained with the ‘sumt’ command in MrBayes 3.2 [31]. We compared 10,000 trees from each set after reducing the trees to taxa shared in all datasets (43 ingroup taxa), using an R script [32] that was based on the Ape package [33].

### Phylogenetic analyses

We performed a number of different Bayesian analyses on parts of the dataset in order to discern the sources of different signals and conflict (Table 3). These analyses include two different alignment options for the ribosomal RNA genes (18S and 28S), molecular-only and total-evidence analyses which included the morphological partition, analyses of the ribosomal and protein-coding genes separately, in the latter case including or excluding third codon positions (third codon positions of CO1 were always excluded), and finally single-gene analyses. The protein-coding genes were also analyzed after translation into amino acids and applying a reversible-jump algorithm to integrate over the fixed-rate amino-acid models implemented in MrBayes. The data matrices and associated consensus trees of all analyses are deposited on TreeBase (URL for reviewers: http://purl.org/phylo/treebase/phylows/study/TB2:S13902?x-access-code=44421680b40bc7867da8bbe7cece2e9c&format=html).

**Table 3:** Overview of phylogenetic analyses.

Analysis	Alignment of rRNA	Data included	nGen, ASDSF (ASDSF-rogue)^1^	Rogue taxa excluded^2^
Total evidence BAli-Phy	BAli-Phy	All^3^, incl. morphology	10M, 0.021 (N/A)	(none)
Total evidence MAFFT	MAFFT	All^3^, incl. morphology	20M, 0.033 (0.031)	Hemiptera, Dermaptera, Tiphiidae, *Scolebythus, Mymaromma, Mymaromella*
Molecular BAli-Phy	BAli-Phy	Molecular data^3^	20M, 0.014 (N/A)	(none)
Molecular MAFFT	MAFFT	Molecular data^3^	30M, 0.014 (0.013)	*Coccobius, Scolia, Mymaromma, Mymaromella, Cephalonomia, Metapolybia, Chrysis*
rRNA	BAli-Phy	18S, 28S	10M, 0.029	(none)
rRNA	MAFFT	18S, 28S	10M, 0.029 (0.027)	*Megalodontes*, Thysanoptera, Hemiptera
Protein coding 12	n/a	CAD^3^, POL^3^, EF1α-F1^3^, EF1α-F1^3^, CO1^3^	25M, 0.036 (0.022)	*Notofenusa, Mymaromma, Pison, Chyphotes, Diplolepis, Myrmecopterina, Rhopalosoma, Ampulex*
Protein coding 123	n/a	CAD, POL, EF1α-F1, EF1α-F1, CO1^3^	50M, 0.040 (0.037)	*Hesperapis*, *Megalyra*, *Psilocharis*
Single gene: CAD	n/a	CAD	10M, 0.019 (0.012)	*Australomymar*, *Myrmecopterina*
Single gene: POL	n/a	POL	20M, 0.018	n/a
Single gene: EF1α-F1	n/a	EF1α-F1	100M, 0.098	n/a
Single gene: EF1α-F2	n/a	EF1α-F2	100M, 0.093	n/a
Single gene: CO1	n/a	CO1^3^	20M, 0.013	n/a

^1^ Number of generations run, average standard deviation of split frequencies ASDSF, before and (in brackets) after removal of the rogue taxa.

^2^ Rogue taxa as identified by RogueNaRok, in descending order of impact according to the raw improvement of support after removal; taxa with at least 0.5 raw improvements are given.

^3^ Third codon position of protein-coding genes excluded from the analyses

All analyses were performed in MrBayes 3.2 [31]. Where applicable, data were partitioned into genes and into first and second versus third codon positions, with substitution models unlinked across partitions. We used model jumping to integrate over the GTR model subspace (“nst=mixed” option in MrBayes) and modeled among-site rate variation with a four-category gamma distribution and a proportion of invariable sites. The morphology partition was modeled using the standard discrete model [34], a “variable” ascertainment bias, and a four-category gamma distribution to model among-character rate variation. For each analysis, we ran four independent runs of four chains each until they had reached topological convergence (ASDSF < 0.05, preferably lower, with 25% of samples discarded as burn-in). In the case of the single-gene analyses of the EF1-α copies, we ran 100 million generations, but ASDSF values remained at about 0.095. In order to capture the uncertainty that might arise through a lack of convergence of the MCMC in these and also in all the other analyses, we scanned the MrBayes output for bipartition frequencies with a standard deviation larger than 0.1 between runs. The corresponding support values are preceded in each tree figure by a question mark, as they might not have been estimated accurately. Samples of all substitution model parameters were adequate in all runs, as judged from the PSRF values being close to 1.0 and effective sample sizes of (usually much) more than 200. In the single-gene analysis of CAD, the outgroup taxa were recovered within Hymenoptera. In order to obtain meaningful signal from this data partition, we repeated the analysis with all outgroups removed, which strongly improved topology convergence.

Although we focus on the Bayesian analyses, we also performed maximum likelihood (ML) analyses for comparison. These analyses were conducted on the combined molecular data and the total-evidence dataset, each under both alignment strategies for the ribosomal partitions. We obtained an estimate for the maximum-likelihood tree from RAxML [35] under a partitioned GTR model for the molecular and the Mk model [34] for the morphological partitions, respectively. To assess support, we performed 1000 bootstrap replicates.

### Rogue taxa identification

We used a new algorithm to search for rogue taxa, i.e., taxa that are highly inconsistent in their phylogenetic placement [36] in our set of Bayesian trees. The algorithm aims to optimize the relative improvement in clade support achieved by removing single or groups of taxa [37]. As input, we used 1,000 evenly spaced trees from the post-burnin phase of the MrBayes tree sets. The program was accessed via the webserver at http://exelixis-lab.org/roguenarok.html under the majority-rule threshold, optimizing overall support, and using maximum dropset sizes of two, three and ten taxa. In all cases, these three dropset sizes led to the same rogue taxa being identified. Rogue taxa associated with a raw improvement (sum of increase in support values) of at least 0.5 (Table 3) were excluded and support values of the consensus tree re-calculated. On the tree graphs, we indicate these new values for all nodes except those directly below the rogue taxon, which show the original value. Rogues (or groups of rogues) are indicated by dashed branches.

## Results

### Alignment and analysis of ribosomal RNA

The MAFFT runs resulted in the shortest alignments, 2027 bp and 5,418 bp for 18S and 28S, respectively. The BAli-Phy alignments are much longer, i.e. 2310 bp and 10,557 bp. The harmonic means of the likelihoods of the Bayesian tree samples retrieved from these alignments (treating gaps as missing data) reflect the alignment lengths, with the longer BAli-Phy alignment reaching a much higher likelihood than the shorter MAFFT alignments (lnL values of -119,599 and -106,394 for the MAFFT and BAli-Phy alignments, respectively). Congruence with the trees retrieved from the protein-coding genes, from the total-evidence analysis that included the BAli-Phy alignment, and even from the total-evidence analysis based on the MAFFT alignment is higher for the BAli-Phy than for the MAFFT alignment (Table 4).

**Table 4:** Resolution and congruence achieved by single partitions.

Partition	Resolution^1^	TE MAFFT^2^	TE BAli-Phy^2^	Protein coding
rRNA MAFFT	95%	0.287	0.264	0.309
rRNA BAli-Phy	95%	0.272	0.221	0.261
CAD	91%	0.339	0.329	0.207
EFF1	79%	0.434	0.440	0.404
EFF2	67%	0.403	0.416	0.419
POL	77%	0.438	0.458	0.415
CO1	74%	0.384	0.381	0.358
Morphology	91%	0.292	0.298	0.362

The Bayesian tree samples obtained from single data partitions are compared to the total-evidence and protein-coding trees using the average standard deviation of split frequencies as a measure of topological distance.

^1^ Resolution of the respective consensus tree after reduction to 43 ingroup taxa present in each dataset, given as the percentage of nodes that were resolved.

^2^ Total-evidence trees

**Figure 2:**
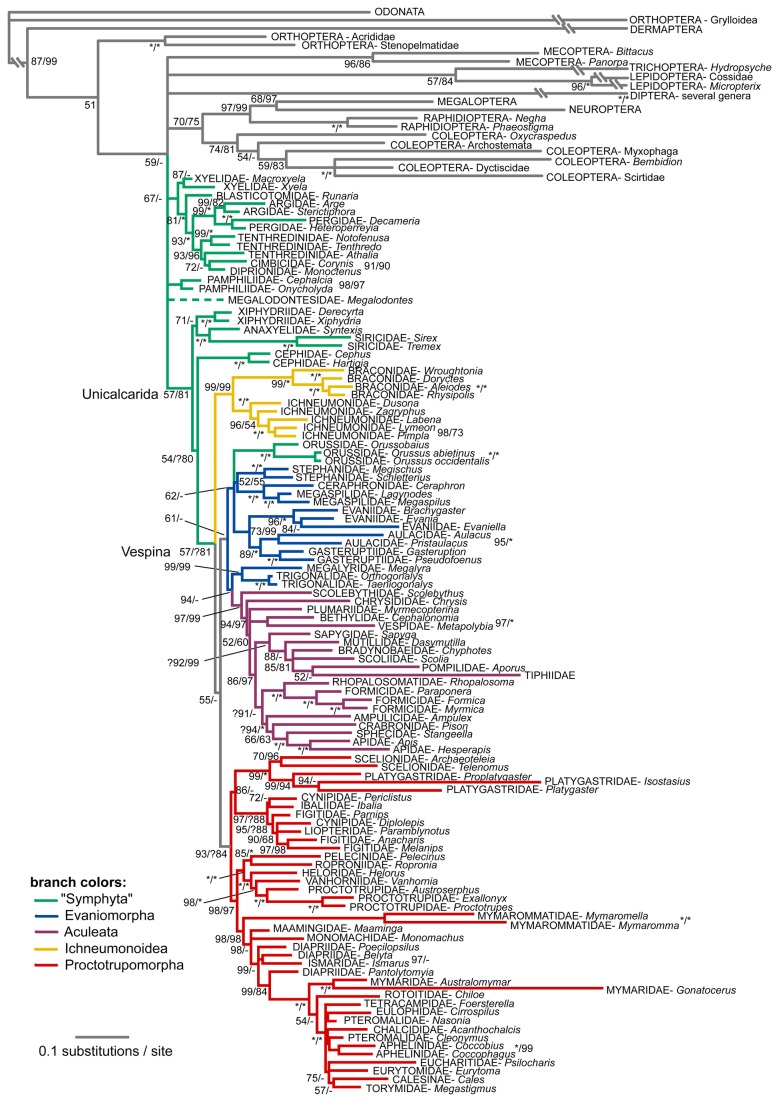
Bayesian tree recovered from the analysis of the two ribosomal genes under the MAFFT alignment. Support values next to the nodes are Bayesian posterior probabilities obtained from the MAFFT and the BAli-Phy alignments, respectively. Asterisks stand for maximal support. Taxa identified as rogues are shown on dashed branches. Very long branches leading to some of the outgroup taxa were compressed in this figure.

The consensus tree retrieved from the rRNA data based on the MAFFT alignment is provided in Figure 2, together with support values from the BAli-Phy alignment. Despite the very different alignment approaches and resulting alignment lengths, the consensus trees do not differ much, but the support values for the MAFFT alignment are usually lower. Interestingly, differences between alignment approaches concern some of the relationships which also differed between the by-eye and secondary structure alignment in the Heraty et al. study [16], i.e. the rooting of the hymenopteran tree and the placement of Orussoidea. Independent of alignment strategy, the rRNA tree is only poorly resolved around the deeper nodes, in contrast to the results from a similar number of base pairs of protein-coding data.

### Phylogeny of *Hymenoptera* as inferred from protein-coding genes

**Figure 3:**
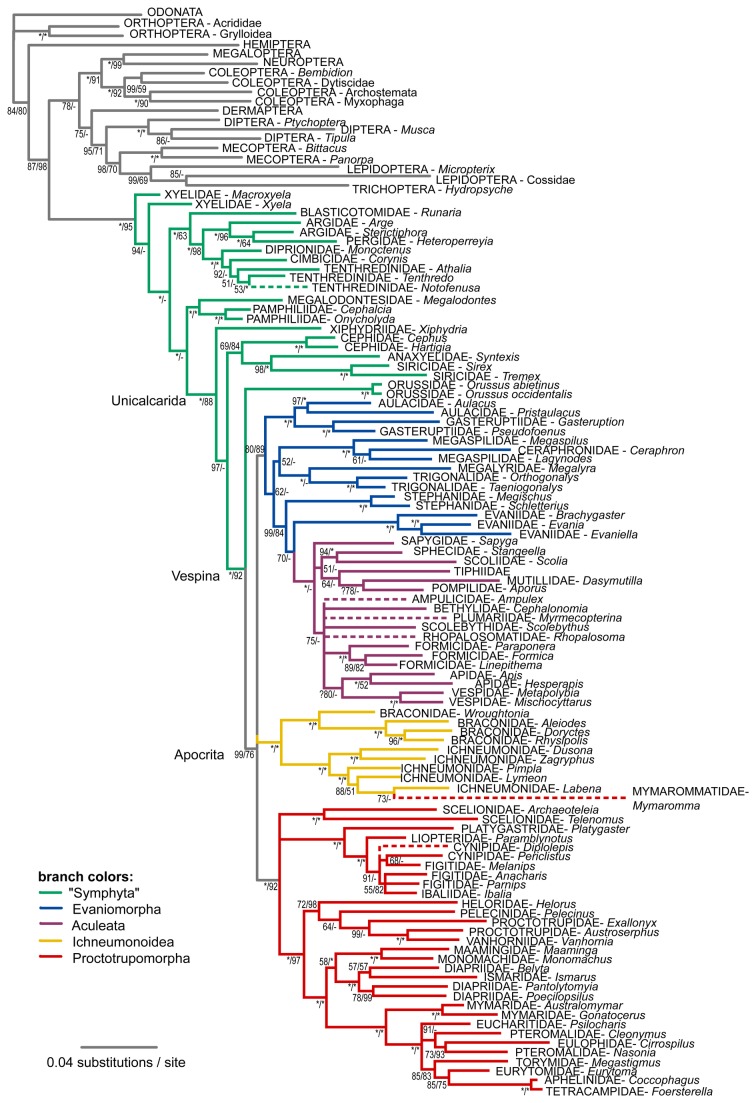
Bayesian tree recovered from the analysis of first and second codon positions of the combined protein-coding genes. Support values next to the nodes are Bayesian posterior probabilities obtained from first and second and from all three codon positions of the nuclear genes, respectively. Asterisks stand for maximal support. Taxa identified as rogues are shown on dashed branches.

Figure 3 shows the tree retrieved from first and second codon positions of the protein-coding genes, along with support values obtained when including third codon positions of the nuclear genes (but not of CO1). The symphytan grade is well resolved, with maximal support on most of the nodes, and with Orussoidea placed firmly as the sister group of Apocrita. Within Apocrita, the Proctotrupomorpha, Ichneumonoidea and (Evaniomorpha + Aculeata) clades are recovered, although only the former two have high support. The relationships among these three are unresolved. In general, superfamilies are recovered, with the exception of paraphyletic Xyeloidea, Evanioidea, Chrysidoidea, Vespoidea, and Platygastroidea. The Xyeloidea are however monophyletic both when including the third codon positions and when analyzing the data as amino acids. As with the rRNA data, resolution is rather low among the evaniomorph superfamilies and within Aculeata. *Mymaromma*, the only representative of the enigmatic Mymarommatoidea, has an incomplete coverage in terms of gene sampling (Table 1). It was identified as a rogue taxon, appearing in different places in the Bayesian tree sample. In the consensus tree, it ended up within Ichneumonoidea, but with low support, and sitting on a very long branch.

Most conflicts with the rRNA tree are weakly supported and/or in areas of the tree which are poorly resolved in both analyses, e.g. the relationships within Evaniomorpha, the placement of Ichneumonoidea and Mymarommatoidea, and the monophyly of Diaprioidea. A notable difference is the sister group of Aculeata, which is the Trigonaloidea + Megalyroidea clade according to the rRNA tree and Evaniidae or Stephanoidea according to the analysis of the protein-coding genes, depending on whether third codon positions were excluded or included.

### Combined molecular results and total-evidence results

**Figure 4:**
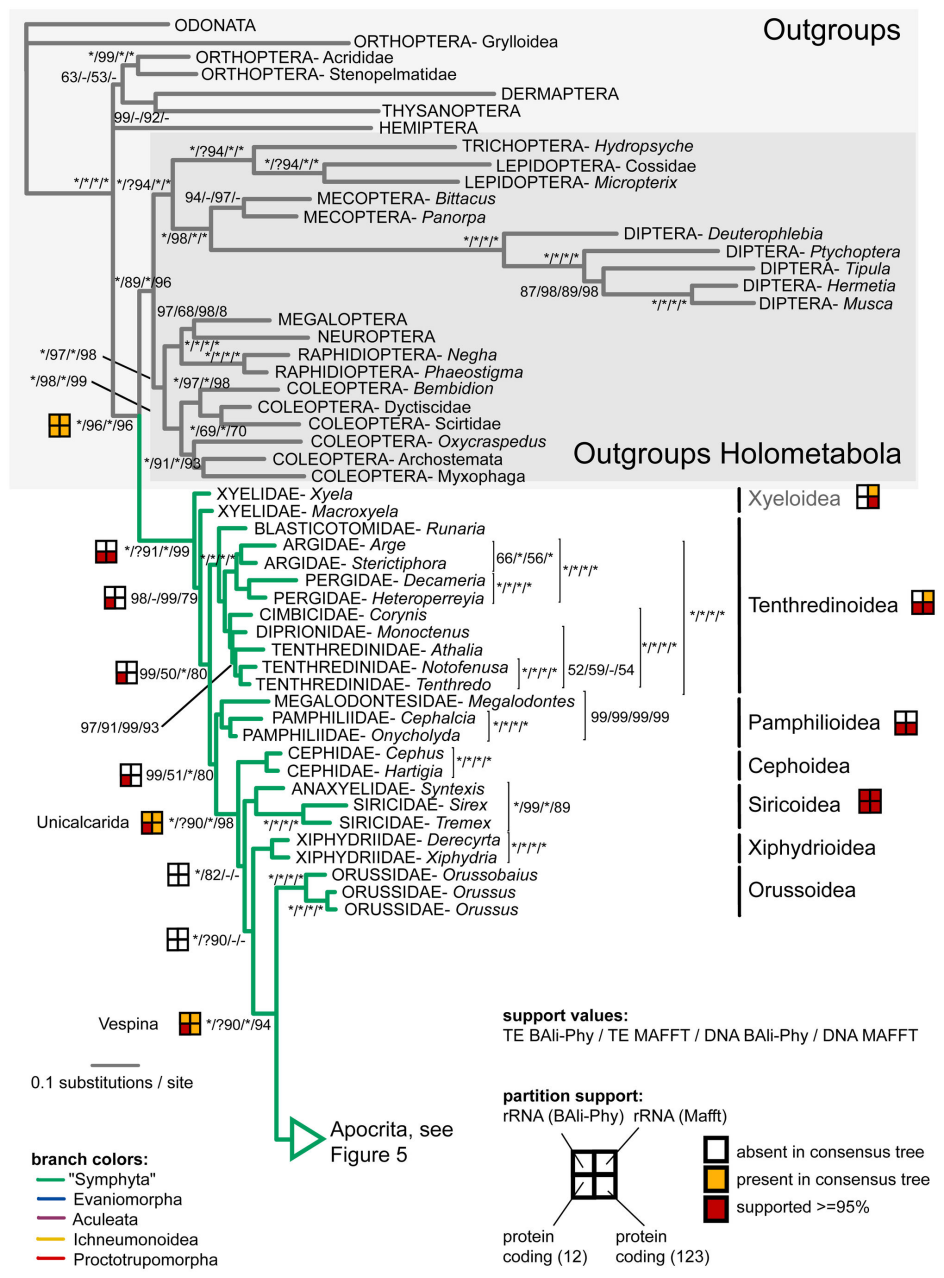
Outgroups and symphytan part of the Bayesian total-evidence tree obtained from the BAli-Phy based alignment. Support values next to nodes indicate the support obtained under either of the two alignment approaches (BAli-Phy and MAFFT) and with morphology included (total evidence, TE), versus the molecular data only, again under both alignment approaches. Asterisks represent maximal support. Symbols indicate support from partitions of the molecular data (see legend). Superfamilies that were not recovered as monophyletic are shown in grey.

**Figure 5:**
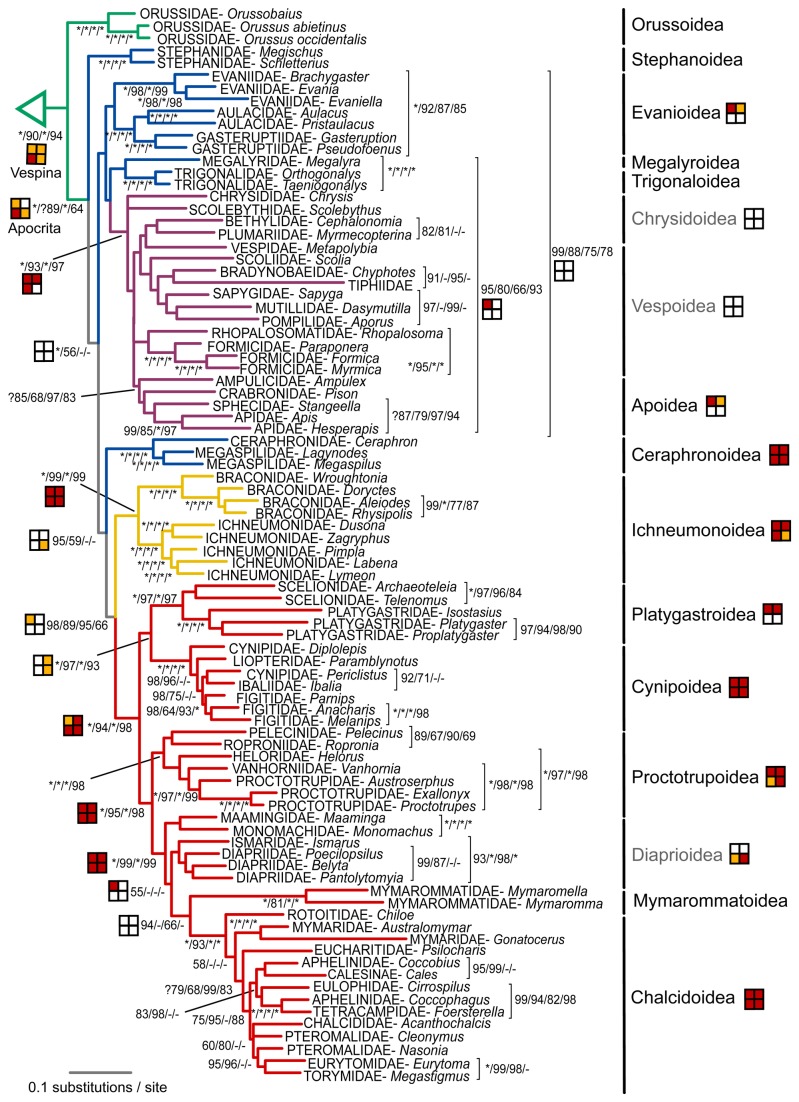
Vespina part of the Bayesian total-evidence tree obtained from the BAli-Phy based alignment. See legend of Figure 4 for details.

The Bayesian total-evidence tree (molecular and morphological data combined) based on the BAli-Phy alignment is given in Figures 4 and 5, including support values from the total-evidence analysis based on the MAFFT-aligned rRNA sequences, and from analogous analyses of the molecular data partition only. The tree also includes symbols summarizing the results from the rRNA data and the protein-coding genes when analyzed separately. Most of the deeper nodes and well-established groupings like the Holometabola, Apocrita, and Aculeata are well supported. When ignoring the uncertain positions of Stephanoidea and Ceraphronoidea, the three large groups within Apocrita — the Ichneumonoidea, Proctotrupomorpha and, with less support, (Evaniomorpha + Aculeata) — are also corroborated. Although most of the proposed superfamilies are recovered as monophyletic, usually with high support, there are several exceptions. First, the most basal superfamily Xyeloidea is paraphyletic, with *Macroxyela* more closely related to the remainder of Hymenoptera. Second, the recently proposed Diaprioidea — including Diapriidae, Maamingidae and Monomachidae — are not supported, although the evidence against its monophyly is weak. Finally, relationships within Aculeata are unstable, and neither Chrysidoidea nor Vespoidea are recovered.

**Figure 6:**
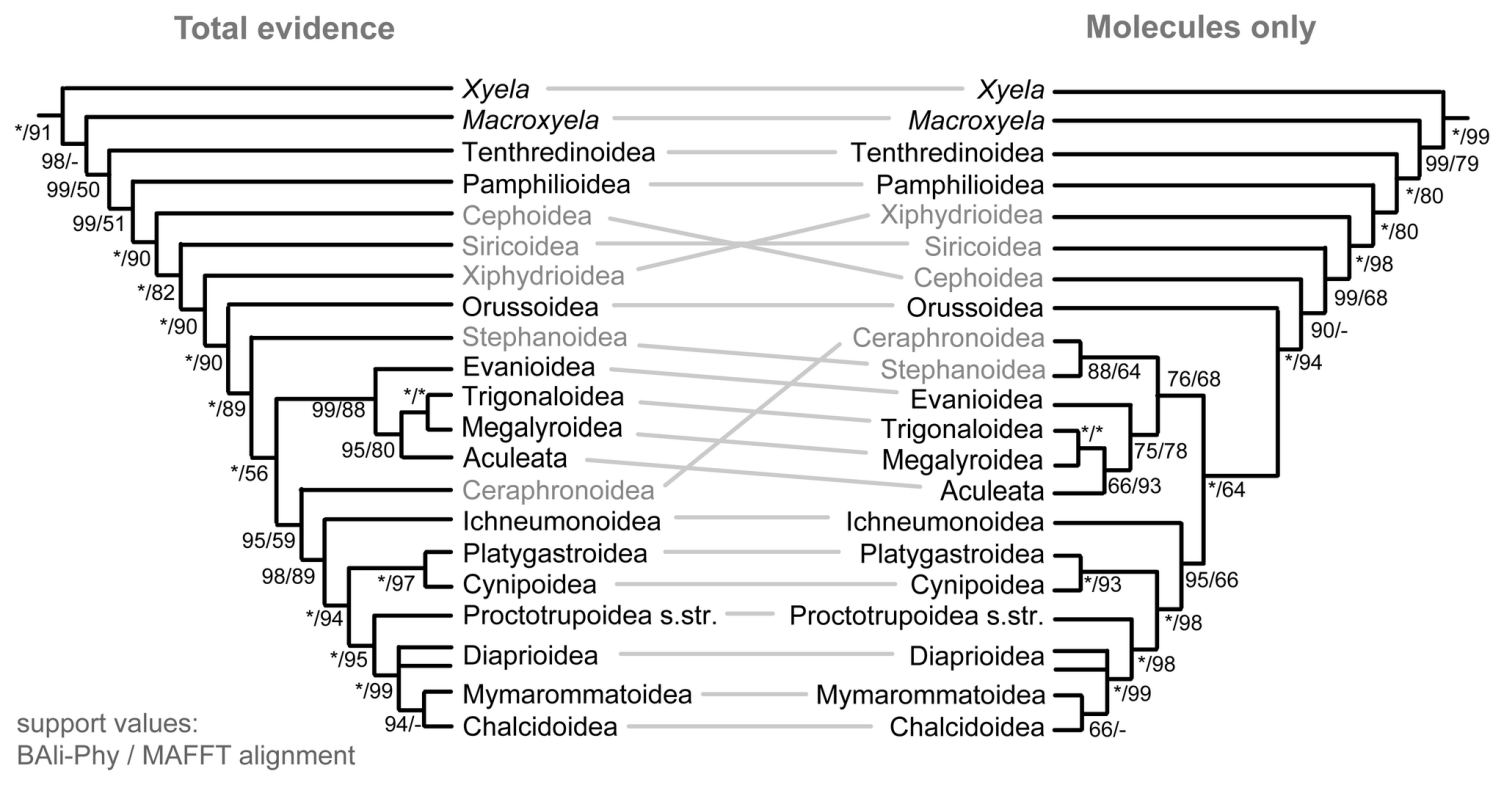
Simplified total-evidence tree based on the combined molecular and morphological data contrasted with the tree obtained from the molecular data alone. Support values are in both cases given for both the BAli-Phy-based and the MAFFT alignment of the rRNA genes, with asterisks representing maximal support. Taxa which assume conflicting positions are shown in grey.

Comparing the total-evidence topology, which included morphological data, to the phylogeny obtained from the molecular data alone, there is considerable congruence, but also two areas where the morphological data have the power to change the molecular results (Figure 6). First, the grade of woodwasps (Siricoidea, Xiphydrioidea and Cephoidea) leading to the Vespina (Apocrita + Orussoidea) is fully reversed in the two analyses, with the sequence Cephoidea – Siricoidea – Xiphydrioidea – Vespina supported by the former, and Xiphydrioidea – Siricoidea – Cephoidea – Vespina by the latter. The molecular signal is fairly strong in the BAli-Phy alignment but weaker in the MAFFT alignment, showing that there is some alignment-dependent signal from the ribosomal sequences. The second example concerns the positions of Stephanoidea and Ceraphronoidea within the Apocrita but involves relationships that are less well supported.

Maximum likelihood estimates based on both the combined molecular and the total-evidence datasets are given in Figures S1 and S2, with bootstrap support values obtained under both the MAFFT and the BAli-Phy alignment approaches for rRNA. The ML trees are similar to those obtained from the Bayesian analyses, but differ with respect to the placement of the hymenopteran root, which is between Tenthredinoidea and the remaining hymenopterans in the total-evidence and between a monophyletic Xyeloidea + Tenthredinoidea + Pamphilioidea and Unicalcarida in the molecular analysis. Furthermore, the total-evidence analyses did not recover a monophyletic Evaniomorpha, but the conflicting nodes were associated with very low bootstrap support.

### Phylogenetic signal in different data partitions

In order to assess the contribution of the different genes and of morphology, we investigate patterns of variation, resolution of the single-gene or single-partition consensus trees, and their congruence with trees derived from other data partitions. Table 2 summarizes some basic properties of the molecular data by gene and by gene partitions. The ribosomal genes and the third codon positions of the two EF1-α copies showed no to moderate GC-biases (up to 61.5% in the third codon positions of EF1-α F1), whereas CO1 had moderate to strong AT bias (62% and 90% for first plus second and third codon positions, respectively), as is the rule for mitochondrial genes in Hymenoptera [38]. All third codon positions of the protein coding genes are heavily saturated, while there appears to be a favorable signal-to-noise ratio in the ribosomal genes and at first and second codon positions of CAD and CO1 (Figure 1). The first and second codon positions of the other three genes (POL, EF1-α F1, EF1-α F2) show comparatively little variation.

Resolution of the single-partition consensus trees varies strongly (employing the majority rule criterion). Table 4 shows the percentage of resolved nodes after reducing each tree to the 43 ingroup taxa common to all datasets. The rRNA data resolved 95% of nodes irrespective of alignment approach, and morphology and the CAD gene each reached 91%. The other single genes lag behind at 67% to 79%. The ranking of partitions is very similar when not only the 43 completely sampled ingroup taxa, but all taxa available per partition are included, with the difference that CAD now outperforms the MAFFT-aligned rRNA data. A similar picture appears when comparing the topological distances between trees obtained from the single-gene analyses to the protein-coding and total-evidence trees (Table 4). The rRNA data and the CAD gene consistently rank highest, followed by morphology and CO1, while POL and the two EF1-α copies result in more conflicting topologies [22].

## Discussion

### Objective and semi-objective alignments of ribosomal DNA sequences

Arguably the best approach to phylogenetic inference based on ribosomal sequences is to analyze unaligned sequences directly. There are several methods that simultaneously estimate alignment and phylogeny: POY in the parsimony framework [39], and ALIFRITZ [40], BAli-Phy [19] and Luntner et al. [18] in a Bayesian setting. These approaches make use of the information present in gaps when reconstructing the phylogeny, which can greatly improve phylogenetic inference [41]. The Bayesian approaches are particularly compelling in that they integrate over alignment uncertainty when reconstructing phylogenetic relationships. Unfortunately, they are still too computationally complex and converge too slowly to be applicable to most empirical datasets.

In addition to the entirely objective alignment approach using MAFFT, we attempted Bayesian analysis of our unaligned data. However, we had to split our dataset both by taxa and sites in order to reach even marginally acceptable convergence on topology and the parameters of the indel model. Merging the MAP sub-alignments and analyzing the composite matrix in a traditional two-step procedure meant we had to forego the possibility of using the information in the indels and integrating over alignment uncertainty. Nonetheless, our partitioned Bayesian method compared favorably to the MAFFT alignment method, as indicated by higher support values and higher congruence with the protein-coding tree.

Although largely objective, our partitioned Bayesian method does involve human decisions on how to decompose the alignment by taxa and by sites. Splitting by taxa has the greatest potential to bias the results. Difficult sequence positions might be aligned in a different manner in the different sub-problems, and the merging of the sub-alignments by OPAL [28] might not be able to resolve such conflict and thus lead to an exaggerated alignment similarity between taxa that were aligned in the same batch. We minimized such problems by basing the decomposition on results from the analysis of the protein-coding genes. We also searched the final results for potential alignment-induced biases. Nodes that might be involved were at the bases of i) all Hymenoptera, ii) Apocrita, iii) Proctotrupomorpha, and iv) all non-proctotrupomorph apocritans. The first three nodes are present in both the trees derived from the BAli-Phy aligned sequences and those resulting from the MAFFT alignment. The fourth group was not recovered in either analysis. In fact, the grouping of Ichneumonoidea with Proctotrupomorpha instead of with Evaniomorpha plus Aculeata, across alignment decomposition lines, was even retrieved with higher support in the BAli-Phy than in the MAFFT analyses (Figure 2). The apparent absence of decomposition-induced artifacts, the fact that clade support values were almost always higher in the BAli-Phy than in the MAFFT analysis, and the higher congruence of the tree sample obtained from the BAli-Phy alignment with the trees from the protein-coding genes indicate that splitting the alignment problem based on a few explicit and well-grounded assumptions about relationships may be a good general strategy for improving alignment quality.

Several candidate alignment artifacts were identified based on a comparison of the by-eye and secondary-structure alignments of Heraty et al. [16], and by comparison with the results from the protein-coding sequences. These include the monophyly of Xyeloidea and Evanioidea, and the placement of Orussoidea among Evaniomorpha. If they were artifacts of a subjective alignment in the previous analyses of ribosomal data, they should disappear in our analyses of objective alignments. However, all these signals were clearly present in our re-analyses of the ribosomal data (Figure 2), even though the support values are generally lower, indicating that subjective bias has possibly augmented these signals.

### Implications for the hymenopteran tree of life

Our analyses recover a large part of the higher-level phylogeny of Hymenoptera with high support and strong corroboration from independent data sources. Many of these relationships have been uncontroversial at least in the more recent past, e.g. the monophyly of the Unicalcarida (Hymenoptera without Xyeloidea, Tenthredinoidea, and Pamphilioidea) [42], the grouping of Orussoidea with Apocrita, the monophyly of Aculeata and Proctotrupomorpha, and of most of the superfamilies as outlined in Sharkey [12]. More recent suggestions that we could corroborate here with independent protein-coding data include Trigonaloidea + Megalyroidea, core Proctotrupomorpha (Proctotrupomorpha excluding Cynipoidea and Platygastroidea), core Proctotrupoidea (Proctotrupoidea without Diaprioidea), and finally the placement of Aculeata within a paraphyletic Evaniomorpha. These results appear to be robust and will probably pass the test of time. As they were discussed at some length in a previous study [17], we will not go into further detail here, but only discuss equivocal relationships.

**Figure 7:**
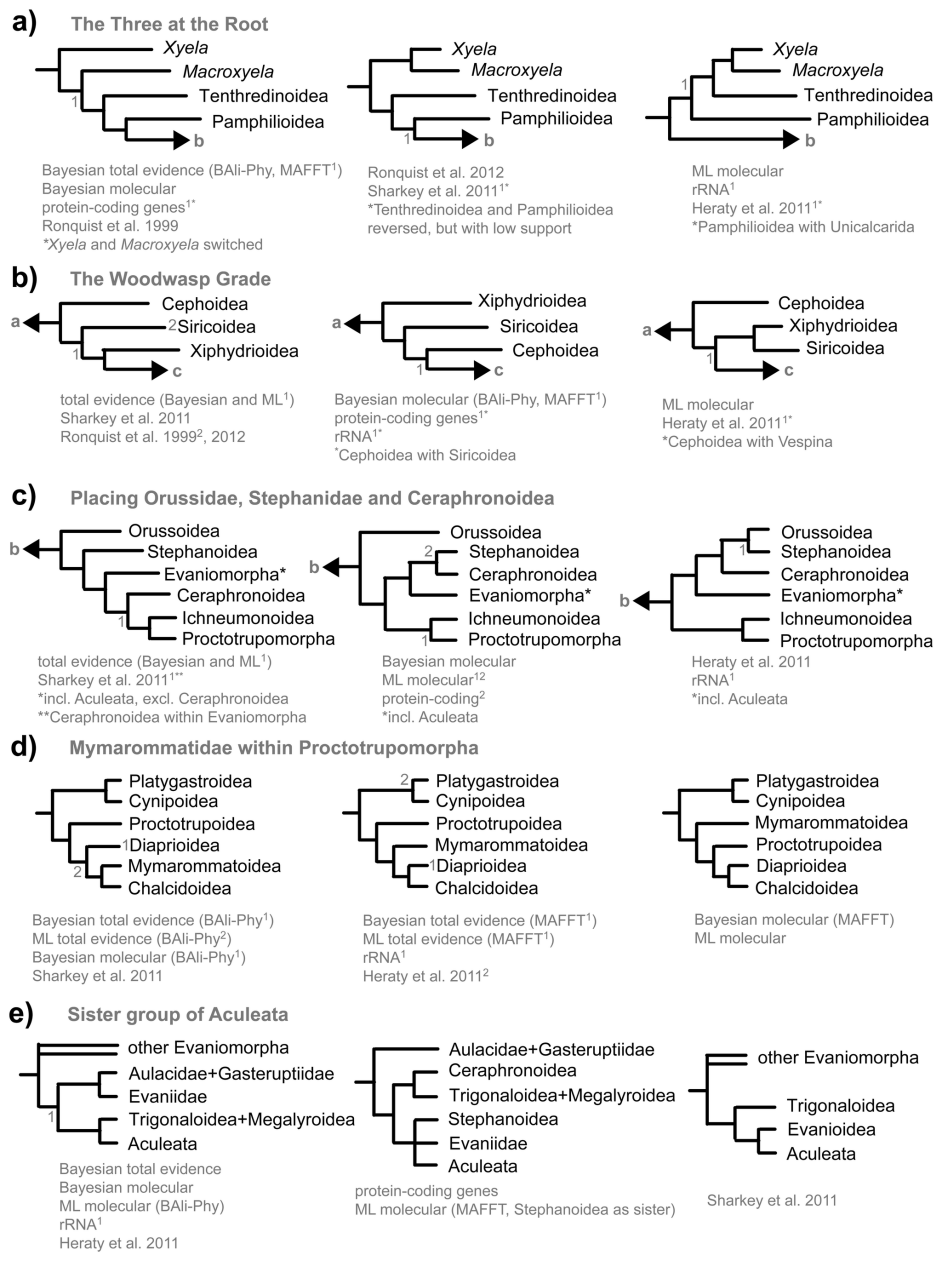
Schematic representation of controversial relationships in high-level phylogenetics of Hymenoptera. Numbers next to nodes and superscripts in the text indicate nodes for which the consensus trees obtained in specific analyses are in conflict with the diagram (see these for details). Numbers next to taxon names stand for non-monophyly of the group. Besides hypotheses derived from our data, we also show selected results from the literature. If a dataset or publication does not appear in one of the cases, then it did not provide any resolution for the relationships in question. In the Heraty et al. (2011) analysis, we refer to the by-eye alignment.

Several parts of the hymenopteran tree remain unresolved and most of these unstable areas include taxa that were also identified as rogue taxa in one or more of the analyses. Rogues can arise due to several reasons, e.g., insufficient gene coverage or particularly long branches. While on average, the twenty taxa identified as rogues did not have a lower number of genes sampled (one missing gene being the average both of the whole dataset and among the rogue taxa), missing data might still be behind the formation of some of the rogues (e.g., Mymarommatoidea, see below). Most of the controversial relationships were also ambiguous in earlier analyses, and might represent difficult phylogenetic histories like rapid radiations (e.g., Aculeata, see below). Figure 7 summarizes the areas of conflict or uncertainty, and we here give a short summary of the evidence for conflicting hypotheses.

#### The three at the root

It has been recognized early on that Xyeloidea, Tenthredinoidea and Pamphilioidea are the three superfamilies closest to the root of Hymenoptera [13,14,42]. However, the relationships among these superfamilies are not resolved. The three competing hypotheses that result from the current and recent analyses are shown in Figure 7a. Most of the controversy boils down to the uncertain placement of the root of the order Hymenoptera – either within Xyeloidea, between Xyeloidea and the remaining Hymenoptera, or between (Xyeloidea + Tenthredinoidea) and the rest. Undoubtedly, the problem is caused to a large extent by the deep roots of the order that probably date back to the Carboniferous [3], in combination with the long branches connecting it to the outgroups [43,44]. As Hymenoptera are probably the sister group to all other holometabolous insects [45,46], only a much denser taxon sampling of both holometabolan and hemimetabolan outgroups could help improve the reconstruction of ancestral sequences, and hence help resolve these deep relationships. Unfortunately, such an approach is limited by the fact that extant outgroups are placed in isolated crown groups of their own and cannot break down the long branch leading to Hymenoptera.

In addition to the rooting problem, it is somewhat unclear whether Tenthredinoidea or Pamphilioidea are more closely related to the Unicalcarida. The former hypothesis was found in the Sharkey et al. [17] total-evidence analysis, but with low support, and in the CAD single-gene analysis, but again with a posterior probability of only 0.53. In contrast, the combined protein-coding genes show maximum support for Pamphilioidea as the sister to Unicalcarida. Morphological evidence is also somewhat equivocal about the placement of the hymenopteran root. Putative synapomorphies that could support a monophyletic Xyeloidea can be found among the mouthparts, e.g. the labral brush, asymmetric mandibles and elongate maxillary palpi [47]. These features are associated with pollen feeding in the adults and are unique within Hymenoptera. In contrast, the long, compound third segment of the antenna which results from the fusion of several flagellomeres might represent a symplesiomorphy, as it is also found in many early fossil hymenopterans and in the tenthredinoid families Blasticotomidae and Argidae [2].

#### The woodwasp grade

The remaining symphytan superfamilies in most analyses form a grade towards Vespina (Orussoidea + Apocrita). The sequence in which they branch off is strongly dependent on the dataset and constitutes one of the two strong conflicts between the morphological and molecular data partitions (Figures 6, 7b). Morphological evidence supporting Xiphydrioidea as sister to Vespina is rather strong; the most convincing proposed synapomorphies for this relationship include a number of characters in the dorsal part of the thorax, e.g., the presence of a transscutal articulation, the reduction of the posterodorsal part of the metapleuron (possibly an incipient step in the formation of the wasp waist in Apocrita), and the loss of a number of thoracic muscles [15]. However, none of the single-gene or various combined molecular datasets supported this relationship, and the combined molecular, protein-coding and CAD single-gene analysis are strongly against. Nevertheless, the signal in the morphological partition is strong enough to resolve this conflict in favor of morphology in the total-evidence analyses.

#### Placement of Stephanoidea and Ceraphronoidea

These two groups are notoriously difficult to place. In the total-evidence analyses, Stephanoidea is placed as the sister-group of all remaining apocritans, a placement that is supported by several morphological, in particular mesosomal, characters [15]. Again, this conflicts with the protein-coding genes, which place stephanids within Evaniomorpha and potentially as the sister clade to Aculeata. The rRNA data do not provide stable resolution around the nodes in question. The situation is complicated by Ceraphronoidea, which assume very differing positions in different analyses, grouping alternatively with Stephanoidea, with Ichneumonoidea, or as sister to Ichneumonoidea plus Proctotrupomorpha. A sister-group relationship between Ceraphronoidea and Megalyroidea, as recovered in Sharkey et al. [17], was never observed here. Morphology does not provide many reliable characters due to the small size of these wasps. Confidence about the placement of Stephanoidea and Ceraphronoidea will depend on additional data, and will help to refine the status of the highly contested Evaniomorpha.

#### Placement of Mymarommatoidea

The placement of this family is complicated by their small size, associated reduction of many otherwise informative morphological character systems, and risk of homoplasy in other character states associated with size. The gene sampling for this taxon was not complete in our analysis, and they came out as a rogue taxon on a very long branch in the protein-coding tree. The rRNA data recover them as the sister group of Diaprioidea plus Chalcidoidea, but the support for this placement disappears in the combined molecular analysis. Including morphology added support for the common interpretation of mymarommatids as the sister group of Chalcidoidea [14,15,17,48], but this was sensitive to the alignment approach. More molecular data is needed to resolve this conflict, especially because of the limitations inherent in the morphological data for these tiny wasps.

#### The sister group of Aculeata

Aculeata are firmly placed within a paraphyletic Evaniomorpha (see next paragraph) in all our analyses. A similar placement was recovered in previous analyses [16,17], and contradicts early hypotheses of a sister-group relationship between Aculeata and Ichnemonoidea [13,14]. Within Evaniomorpha, however, the relationships are highly unstable, and the sister-group of aculeates remains unclear. Although there is some indication that the strongly supported Trigonaloidea + Megalyroidea clade is sister to aculeates, support is weak, alignment-dependent, and contradicted by the analysis of the concatenated protein-coding genes, which favored either Stephanoidea or Evaniidae as the sister group. Given the low resolution both among evaniomorph superfamilies and within Aculeata, a denser taxon sampling within these groups is probably needed to clarify this question.

#### Evaniomorpha

The concept of Evaniomorpha, as originally proposed by Rasnitsyn [13], included the superfamilies Stephanoidea, Ceraphronoidea, Megalyroidea, Trigonaloidea and Evanioidea, while excluding Aculeata. The morphological and fossil evidence supporting this somewhat heterogeneous assemblage has always been weak, and Rasnitsyn himself recently proposed that the Evaniomorpha be restricted to the Evanioidea [49]. The circumscription of Evaniomorpha remains unclear even after our analyses, especially with respect to Stephanoidea and Ceraphronoidea, but it should definitely be revised to include Aculeata if it is retained as a concept defining a major apocritan lineage.

#### Non-monophyletic superfamilies

The superfamilies not recovered as monophyletic in the total-evidence analyses are the following: Xyeloidea, Chrysidoidea, Vespoidea and Diaprioidea. While the Xyeloidea are discussed above, the remaining superfamilies deserve further attention. There are several rather convincing morphological synapomorphies for Chrysidoidea, e.g., the subdivision of the second valvifer of the ovipositor into two articulating parts [50], and their non-monophyly was in fact not strongly supported; rather, the relationships among aculeate families are very poorly resolved in all our analyses, and a much denser taxon and gene sampling is obviously required to address these relationships. The same is true for Vespoidea, although it has been hypothesized previously that they are paraphyletic with respect to Apoidea [7,51].

The superfamily Diaprioidea was suggested by Sharkey [12] to include Diapriidae, Maamingidae and Monomachidae, based on an earlier molecular analysis [52]. While not retrieved in the total-evidence analysis, which instead suggested paraphily with respect to Mymarommatoidea and Chalcidoidea (but with very low support), Diaprioidea are recovered in the CAD single-gene, the protein-coding, and the combined molecular analyses.

Although the Evanioidea were recovered as monophyletic in the total-evidence and combined molecular tree, they were split into Gasteruptiidae + Aulacidae versus Evaniidae in the protein-coding and CAD single-gene analyses. This superfamily may thus deserve more attention, especially given the weak support from morphology, the most striking putative synapomorphy being the attachment of the metasoma high above the hind coxal cavities [e.g. 13,15].

### The future of hymenopteran phylogenetics

Although we present here the most comprehensive study of higher-level hymenopteran relationships to date, many questions of great taxonomic and evolutionary interest remain unresolved; the search for more and better data must thus continue. In the light of the large differences in information content in the genes studied here, it becomes clear that data quality can strongly influence the outcome of studies of deep-level relationships. The performance of CAD [53] was especially outstanding. With less than 1,000 bp, this marker recovered a largely resolved phylogeny of Hymenoptera that was in close agreement with the total-evidence tree. Overall, data partitions that did not show signs of saturation and at the same time included a relatively large number of parsimony-informative sites consistently achieved higher congruence with trees derived from independent and total-evidence partitions. This is in line with recent theoretical and phylogenomic studies, which found a connection between evolutionary rate, saturation, and phylogenetic utility of different markers [54–58]. Data quality might thus play a very important role when it comes to utility for phylogenetic inference, and could render it unnecessary to accumulate huge quantities of data even (or maybe especially) for difficult phylogenetic problems.

On the other hand, the lack of resolution in vital parts of the hymenopteran tree as inferred here from seven genes might simply demonstrate the limits of few-gene approaches. Estimates of the numbers of genes necessary for reliable phylogenetic inference depend strongly on the phylogenetic context and the inference method, but have been suggested to lie around 20 [43,59,60]. Gene sampling for Hymenoptera phylogenetics has until now relied mostly on very few genes, with two exceptions. A study of 24 expressed sequence tags (ESTs) in 10 disparate hymenopteran taxa [61] recovered deep-level relationships which were almost invariably controversial and in conflict with any previous study, e.g. Chalcidoidea placed outside Proctotrupomorpha and a sister-group relationship between the latter and Aculeata. These relationships are likely due to the extremely low taxonomic coverage and potentially also to limited phylogenetic signal in the different markers. Another analysis of phylogenomic proportions made use of all sequence data for Hymenoptera present in Genbank [62]. By developing a bioinformatics pipeline that filtered the vast amount of data for genes with compositional stationarity and defined levels of density and taxonomic overlap, they retrieved about 80,000 sites for 1,100 taxa. The main problem with this dataset was the amount of missing data (more than 98%). The resulting tree had very low resolution, recovered many of the included families as para- or polyphyletic, and placed some taxa in obviously erroneous positions. Nevertheless, some of the superfamilies and undisputed higher-level relationships were recovered in this analysis, which demonstrates the potential of such an approach.

The future of hymenopteran phylogenetics lies in datasets that combine the advantages of each of the afore-mentioned studies, i.e., a dense and balanced taxon sampling [63–65], sufficiently large amounts of molecular data, a careful assessment of the quality of this data [55,66], and appropriate analysis methodology. Only the combination of these is likely to resolve the remaining uncertainties in the evolutionary history of a group that originated hundreds of million years ago and diversified into hundreds of thousands of species.

## Supporting Information

Table S1 **Commented table of primers used in this study.**
(PDF)

Figure S1 **Maximum likelihood tree recovered from the analysis of the combined molecular data (**rRNA MAFFT aligned**). **
Support values next to the nodes (or after species pairs) are bootstrap supports obtained from 1000 replicates based on both the MAFFT and the BAli-Phy alignments, respectively. Asterisks stand for maximal support.
(TIF)

Figure S2 **Maximum likelihood tree recovered from the analysis of the combined molecular and morphological data (**rRNA BAli-Phy aligned**). **
Support values next to the nodes (or after species pairs) are bootstrap supports obtained from 1000 replicates based on both the BAli-Phy and the MAFFT alignments, respectively. Asterisks stand for maximal support.
(TIF)
