# Supplementary material for: The Hymenopteran Tree of Life: Evidence from Protein-Coding Genes and Objectively Aligned Ribosomal Data
Source: PLoS One. 2013 Aug 2;8(8):e69344. doi: 10.1371/journal.pone.0069344 (PMC3732274; doi:10.1371/journal.pone.0069344)
Supplement: Table S1 — Commented table of primers used in this study. (PDF) [file pone.0069344.s001.pdf]

Supplementary Table 1. Commented table of primers used in this study (Klopfstein et al., The hymenopteran tree of life: evidence from protein-coding genes and objectively aligned ribosomal data).

Gene	Fragment, direction	Primer name	Sequence	Comments
**CAD**[1]	1, forward	ap787for2	TGCTTYGARCCDAGYCTHGATTAYTGCAD	Spanning two introns when combined with 1098rev3
		CADfor2	GCYTGYTTYGARCCNAGYCTNGA	Spanning two introns when combined with 1098rev3
		CADskFor1	GCGTNGTSAARATNCCRMGRTGGG	This study; spanning two introns when combined with 1098rev3
		CADfor11	TGGGATYTNNGYARRTTYCA	
		CADfor9s	ATGAARAGYGTNGGNGARGTRATGGC	Shorter fragment: very good success with taxa that have long introns in CAD (e.g. Ichneumonidae, some Aculeata, etc.)
	1, reverse	1098rev3	ATATTRTTKGGNARYTGDCCKCCC	
		CADskRev1a	CRATRACCATNGTRTARCCNCCNGG	This study; spans only one intron when combined with ap787for2, CADfor2, CADskFor1, or no intron when combined with CADfor9s
	2, forward	apCADfor3	CTCHGTKGARTTYGATTGGTGYGC	Combine with either of the two next ones, includes one intron
		CADfor4	GTKGARTTYGATTGGTGYGCNGT	
	2, reverse	apCADrev3a	CARGGRTARCCRACYTCYTCRCAAAATTC	
		CADrev4	CANGGRTARCCRACYTCNTCGCA	
**RNA pol II**[2]	forward	polfor2	TGGGAYGSYAAAATGCCKCAACC	
		polfor5	TGATGAAYNTKYTNATGT	
		polceraf1	GTAAAYATGATAAGACANCACAG	After first intron in Ceraphronoidea and Cynipoidea
	reverse	polrev2	TTYACAGCAGTATCRATRAGACCTT	
		polrev5	GCAGTATCRATNAGACCTTCNCG	

[1] The stretch of CAD amplified here contained three introns. These were present in most taxa, but some appear to be intron-free (e.g. Braconidae). Lenghts of introns varied strongly, and long introns often hindered successful amplification of more than one exon.

[2] The RNA pol II portion amplified here was intron-free in most taxa, but introns were found in Ceraphronoidea, Cynipoidea, Scelionidae and some Chalcidoidea.
