# Supplementary figures and images for: The Hymenopteran Tree of Life: Evidence from Protein-Coding Genes and Objectively Aligned Ribosomal Data
Source: PLoS One. 2013 Aug 2;8(8):e69344. doi: 10.1371/journal.pone.0069344 (PMC3732274; doi:10.1371/journal.pone.0069344)

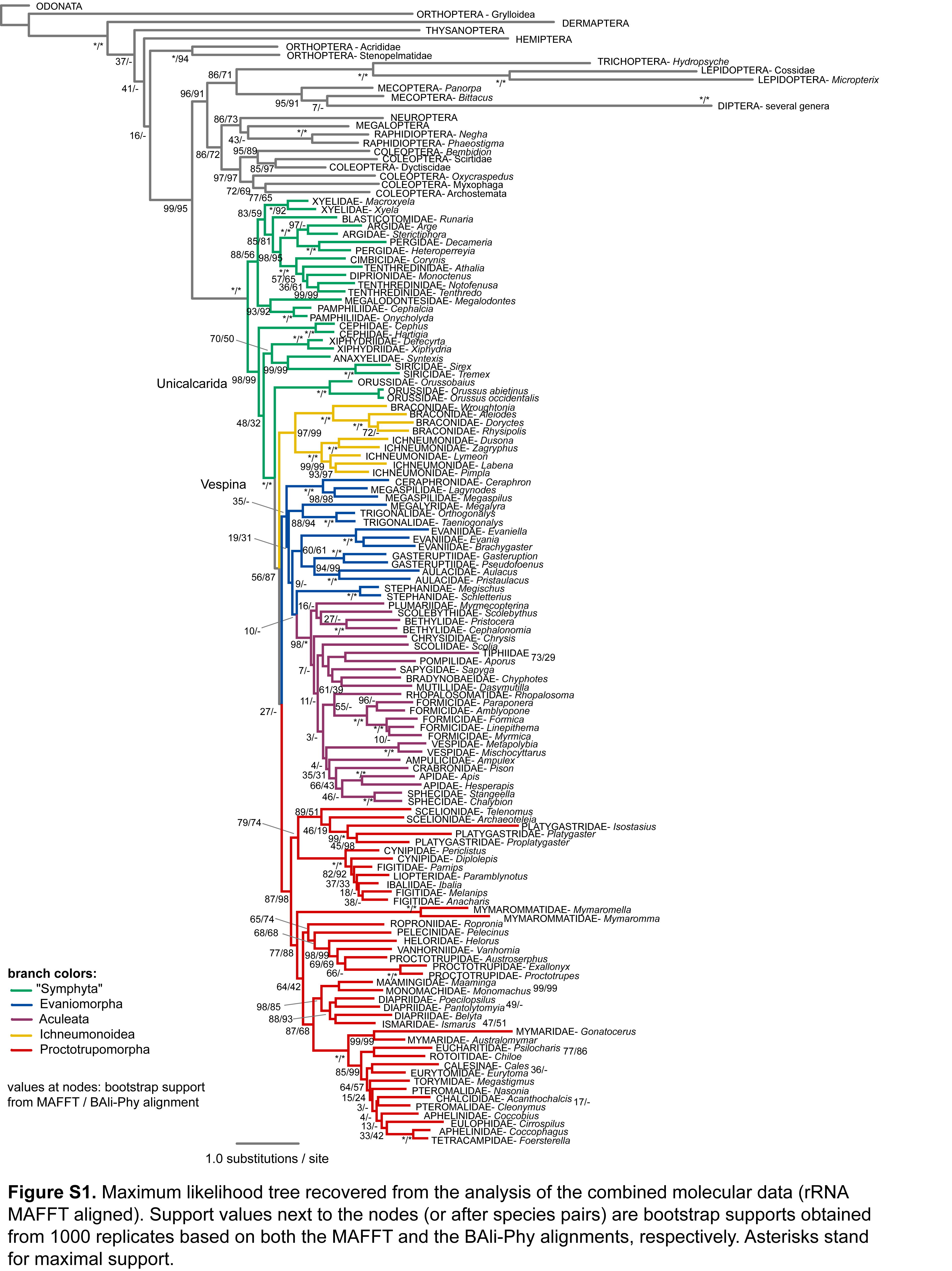

Supplement: Figure S1 — Maximum likelihood tree recovered from the analysis of the combined molecular data (rRNA MAFFT aligned). Support values next to the nodes (or after species pairs) are bootstrap supports obtained from 1000 replicates based on both the MAFFT and the BAli-Phy alignments, respectively. Asterisks stand for maximal support. (TIF) [file pone.0069344.s002.tif]

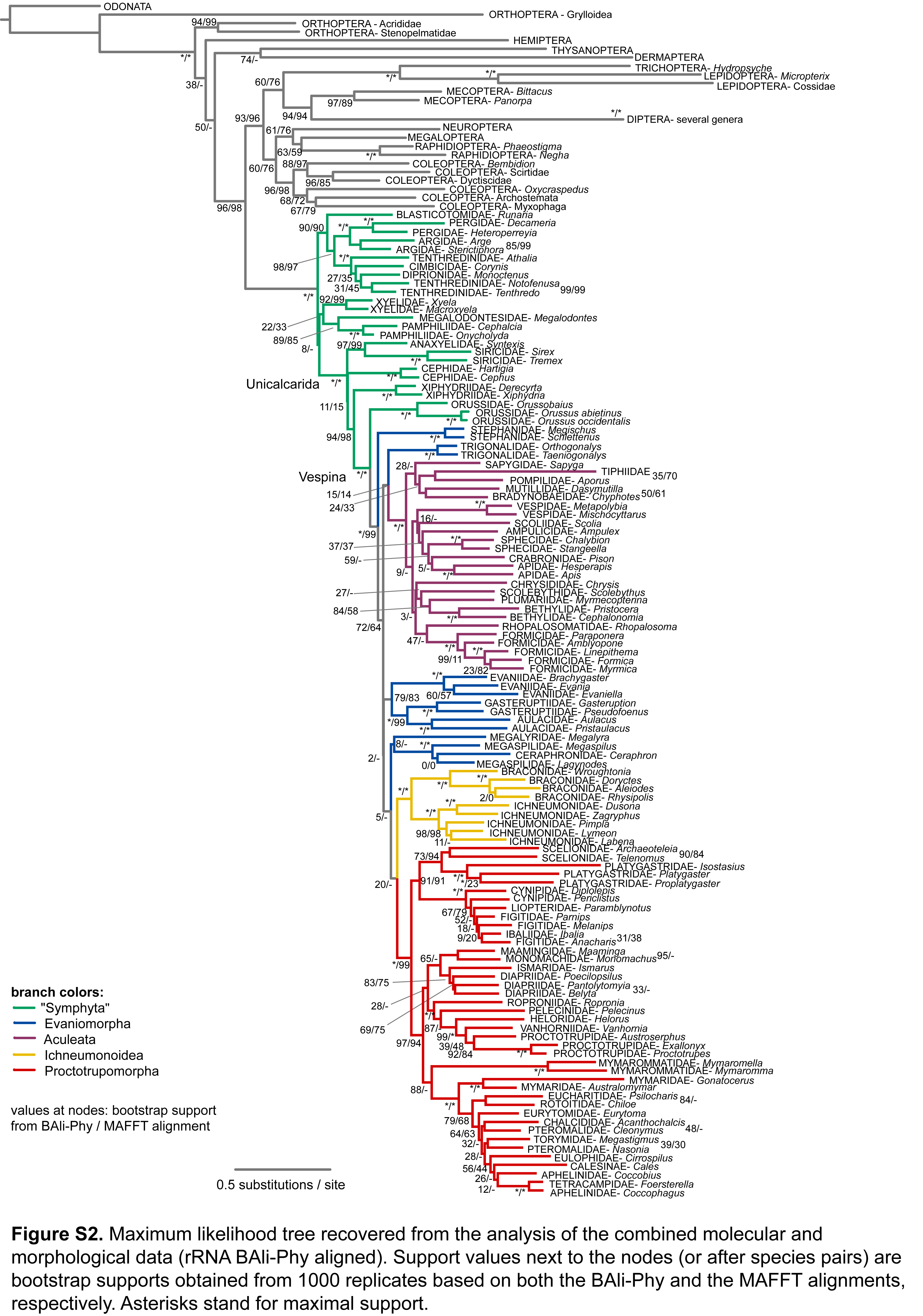

Supplement: Figure S2 — Maximum likelihood tree recovered from the analysis of the combined molecular and morphological data (rRNA BAli-Phy aligned). Support values next to the nodes (or after species pairs) are bootstrap supports obtained from 1000 replicates based on both the BAli-Phy and the MAFFT alignments, respectively. Asterisks stand for maximal support. (TIF) [file pone.0069344.s003.tif]
